# Modeling Macrophage Polarization and Its Effect on Cancer Treatment Success

**DOI:** 10.4236/oji.2018.82004

**Published:** 2018-06-29

**Authors:** Valentin Morales, Luis Soto-Ortiz

**Affiliations:** 1 Department of Engineering and Technologies, East Los Angeles College, Monterey Park, USA; 2 Department of Mathematics, East Los Angeles College, Monterey Park, USA

**Keywords:** Macrophage Polarization, Feedback Loops, Immunosuppression, Mathematical Modeling, Cancer Therapy

## Abstract

Positive feedback loops drive immune cell polarization toward a pro-tumor phenotype that accentuates immunosuppression and tumor angiogenesis. This phenotypic switch leads to the escape of cancer cells from immune destruction. These positive feedback loops are generated by cytokines such as TGF-*β*, Interleukin-10 and Interleukin-4, which are responsible for the polarization of monocytes and M1 macrophages into pro-tumor M2 macrophages, and the polarization of naive helper T cells intopro-tumor Th2 cells. In this article, we present a deterministic ordinary differential equation (ODE) model that includes key cellular interactions and cytokine signaling pathways that lead to immune cell polarization in the tumor microenvironment. The model was used to simulate various cancer treatments in silico. We identified combination therapies that consist of M1 macrophages or Th1 helper cells, coupled with an anti-angiogenic treatment, that are robust with respect to immune response strength, initial tumor size and treatment resistance. We also identified IL-4 and IL-10 as the targets that should be neutralized in order to make these combination treatments robust with respect to immune cell polarization. The model simulations confirmed a hypothesis based on published experimental evidence that a polarization into the M1 and Th1 phenotypes to increase the M1-to-M2 and Th1-to-Th2 ratios plays a significant role in treatment success. Our results highlight the importance of immune cell reprogramming as a viable strategy to eradicate a highly vascularized tumor when the strength of the immune response is characteristically weak and cell polarization to the pro-tumor phenotype has occurred.

## 1. Introduction

### 1.1. Immune Cell Polarization

The differentiation and polarization of certain immune cells into pro-inflammatory and anti-inflammatory cells confer the immune system with versatility to exhibit a strong, but controlled, response against invading pathogens and foreign antigens. This is made possible by the initial generation of a strong inflammatory response that is subsequently regulated and attenuated by an anti-inflammatory response once the invading agents have been destroyed. A phenotypic switch by immune cells during an infection and after its resolution makes it possible for the immune system to regulate its own activity and return to a state of homeostasis [1].

When healthy cells mutate and become cancerous, immune cells such as natural killer cells and cytotoxic T lymphocytes (CTL) try to eliminate these anomalous cells. They do so by infiltrating the tumor site and releasing proteins that destroy the cancer cell membrane (Perforin), and release enzymes that lead to cancer cell apoptosis (Granzyme B) [2]. Other immune cells, such as macrophages, are capable of directly phagocytosing the cancer cells. Macrophages that attempt to kill tumor cells via contact-dependent mechanisms or through molecule secretion are classified as M1 macrophages. M2 macrophages tend to exhibit a pro-tumor phenotype characterized by the release of immunosuppressive and angiogenic agents that allow a tumor to survive and grow. It is believed that the M1-to-M2 macrophage concentration ratio can tip the balance in favor of tumor destruction if this ratio is high enough, or in favor of tumor survival if this ratio is close to zero [3]. Macrophage repolarization rates influence the M1-to-M2 ratio which, in turn, could be used to predict tumor size [4]. Similarly, naive helper T cells differentiate into anti-tumor Th1 helper cells or into pro-tumor Th2 helper cells depending on the relative concentrations of anti-tumor and pro-tumor cytokines found in the tumor microenvironment [5]. Consequently, the Th1-to-Th2 helper cell ratio also affects the likelihood of tumor destruction or survival [6]. A high Th1-to-Th2 ratio increases the likelihood of tumor destruction, whereas a ratio close to zero increases the likelihood of tumor survival [7].

Experimental work has shown that it is possible to reprogram M2 macrophages to develop an M1 phenotype by increasing the environmental concentration of anti-tumor cytokines. Monocytes and naïve helper T cells develop an M1 and Th1 phenotype, respectively, if the concentration of anti-tumor cytokine-sINF-*γ* and TNF-*α* is high. Given this phenotypic plasticity of immune cells, it is no surprise that cancer cells have evolved ways to hijack the mechanisms of cell polarization for their own advantage. Cancer cells can escape immune destruction by releasing pro-tumor cytokines, such as TGF-*β*, that decrease the M1-to-M2 macrophage ratio and the Th1-to-Th2 helper cell ratio. What makes this hijacking relevant, in the context of cancer treatments, is that macrophages and helper T cells are found in the tumor site in relatively high concentrations [8] [9] [10] [11]. The high proportion of M2 macrophages and Th2 helper cells in the tumor microenvironment makes them an important target of cancer therapies [12] [13] [14] [15] [16].

### 1.2. Positive Feedback Loops Perpetuate Cell Polarization

A tumor is a complex dynamical system, and its survival depends on a diverse set of signaling networks characterized by cytokine-driven positive feedback loops that can reinforce the anti-tumor phenotype or the pro-tumor phenotype of tumor-infiltrating immune cells. For example, M1 macrophages secrete IL-12 which leads to the differentiation of immature helper T cells into Th1 cells. Th1 cells secrete IFN-ϒ which reinforces the M1 macrophage phenotype. This positive feedback loop perpetuates the M1 and Th1 anti-tumor polarization of these cells, which can lead to tumor destruction. On the other hand, M2 macrophages secrete IL-4 and IL-6 [17], [18] which lead to the differentiation of immature helper T cells into Th2 cells. Th2 cells secrete IL-4 [19] which reinforces the M2 macrophage phenotype. This positive feedback loop perpetuates the M2 and Th2 pro-tumor polarization, leading to tumor escape.

**Figure 1.**
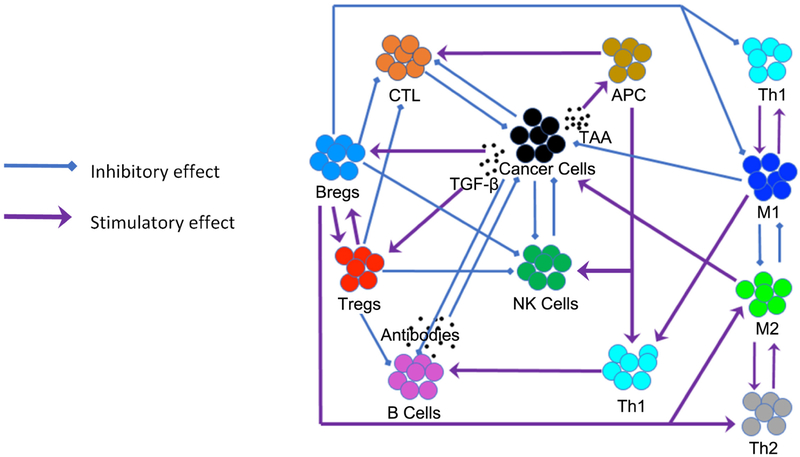
Illustration of the tumor-immune system interactions.

More complex immune cell interactions exist. M2 macrophages and Th2 cells secrete TGF-*β* which converts naïve helper T cells into pro-tumor regulatory T cells (Tregs) [20] and B cells into pro-tumor regulatory B cells (Bregs). Tregs and Bregs secrete IL-10 and TGF-*β*, which reinforces the Th2 phenotype. Th2 cells then reinforce the M2 phenotype and, as a result, the pro-tumor activity of all these cells is perpetuated. Moreover, by releasing IL-10 and TGF-*β*, Tregs and Bregs reinforce each other’s pro-tumor characteristics. The presence of positive feedback loops can lead to switch-like dynamical behavior and bi-stability [21], [22]. Self-perpetuating positive feedback loops and switch-like bi-stability can make a tumor robust with respect to external perturbations, such as a cancer treatment. Positive feedback loops generate attracting dynamical states from which it is difficult to escape without a strong external perturbation. It is for this reason that combination treatments have become a promising approach to treat cancer. Figure 1 illustrates the complex interactions between cancer cells and the immune system, between the immune cells, and the positive feedback loops that characterize such interactions. A thorough review of these interactions can be found in [23].

This article is organized as follows. In Section 2 we describe the mathematical model that was formulated to investigate potential ways to break up pro-tumor feedback loops that lead to treatment failure. We also used the model to assess the relative effectiveness of various combination treatments. In Section 3 we describe the predictions of the model, including the combination treatments that were found to be robust with respect to the level of immune response strength, the tumor size at the start of treatment, treatment resistance, and immune cell polarization. In Section 4 we discuss the implications of the model predictions and comment on ways that the model can be improved. We conclude by highlighting the importance of increasing the M1-to-M2 ratio and the Th1-to-Th2 ratio to boost the anti-tumor immune response, in conjunction with a reduction of TGF-*β*-driven angiogenesis. This approach is predicted to lead to treatment synergy and robustness, and to the effective disruption of the pro-tumor cytokine-driven signaling networks that lead to tumor survival.

## 2. Mathematical Model

### 2.1. Model Description

In [24], Fernandez and Soto-Ortiz expanded an ordinary differential equation (ODE) model of drug resistance [25] to include tumor angiogenesis stimulated by TGF-*β* and immunosuppression exerted by regulatory T cells. Our model expands [24] to include cytokine-driven feedback loops that lead to the polarization of macrophages and helper T cells into anti-tumor cells or pro-tumor cells. The model includes ODEs describing the change in the concentration of M1 and M2 macrophages, Th1 and Th2 helper cells, and the concentration of anti-tumor cytokines IL-1*β*, IL-12, TNF-*α* and IFN-*γ* and pro-tumor cytokines IL-4, IL-10, IFN-*α* and TGF-*β*. We modeled IL-2 as an anti-tumor and pro-tumor cytokine because IL-2 can stimulate NK cells [26], CTL [27] and Tregs [28].

The ODE equations were coded in Scilab (http://www.scilab.org/) and were solved by using a built-in 4th-order explicit Runge-Kutta method with fixed step size. This numerical method has a fast rate of convergence of O(h^4^) and guarantees a stable computation time. Many of the model parameter values were obtained from published literature pertaining to experimental or mathematical modeling work involving various murine and human cancers, as well as infection models that describe a pathogen-immune system interaction that is similar to a tumor-immune system interaction.

It is known that the phenotypes of macrophages vary widely and, thus, they cannot be classified as being purely anti-tumor or purely pro-tumor [29]. Macrophages tend to exhibit a heterogeneity of phenotypes that sometimes overlap. To simplify the development and analysis of the model, we assumed that all the differentiated macrophages have an anti-tumor or pro-tumor phenotype, and that monocytes can differentiate into M1 or M2 macrophages. We also assumed that when an anti-tumor or pro-tumor macrophage repolarizes by changing its phenotype, it immediately adopts the opposite phenotype without delay and without passing through an intermediate state.

The Th1 and Th2 phenotypes of helper T cells tend to be terminally-differentiated states [23]. Repolarization between the Th1 and Th2 states seldom occurs under natural conditions. Experimentally, Th1 and Th2 cells can be repolarized under specific experimental conditions [30]. Hence, our model assumes that once naive helper T cells differentiate and polarize into a Th1 or Th2 state, they maintain that terminal phenotype throughout their existence. The Th1-to-Th2 cell ratio cannot be modulated directly through repolarization of Th1 or Th2 cells. However, the Th1-to-Th2 cell ratio can be modulated indirectly through macrophage repolarization which then polarizes naïve helper T cells, or by direct infusion of polarized helper T cells.

### 2.2. Model Variables and Initial Conditions

The equations of the model are presented in Appendix A. The ODEs that describe the population dynamics of treatment-sensitive and treatment-resistant cancer cells include terms that represent cancer cell destruction by NK cells, CTL, M1 macrophages and Th1 helper T cells. Chemotherapy kills cancer cells and immune system cells. We did not incorporate an anti-tumor humoral response. In the cytokine equations, we included terms that represent antagonistic interactions between certain cytokines. For example, an increase in the concentration of IL-4 and IL-10 decreases the production rate of IL-12 by M1 macrophages and by Th1 helper cells. Similarly, an increase in the concentration of IL-12 decreases the production rate of IL-4 by M2 macrophages and by Th2 helper cells. Antagonistic effects between IFN-*α* and TNF-*α*, and between IFN-*α* and IFN-*γ* were also included. Supplementary Table S1 in Appendix B lists the definitions of the model variables and their units.

To investigate the joint effect of immunosuppression, tumor angiogenesis and immune cell polarization on treatment success, we considered three different scenarios at the start of treatment. These scenarios are listed below in order from most favorable to least favorable:

1. Low immunosuppression, low angiogenesis and no pro-tumor polarization.
2. High immunosuppression, high angiogenesis and no pro-tumor polarization.
3. High immunosuppression, high angiogenesis and high pro-tumor polarization.

To simulate scenario 1, the initial conditions were chosen by assuming a low concentration of Tregs, TGF-*β* and activated endothelial cells, and by assuming that no M2 macrophages and no Th2 cells are initially present. To simulate scenario 2, the initial conditions were chosen by assuming a high concentration of Tregs, TGF-*β* and activated endothelial cells, but no M2 macrophages and no Th2 cells are initially present. To simulate scenario 3, the initial conditions were chosen to reflect a high concentration of Tregs, TGF-*β*, activated endothelial cells, M2 macrophages and Th2 cells. Supplementary Table S2 in Appendix B lists the initial conditions that represent each of these scenarios. Many of these initial conditions are the same that were used in [24]. For the case of an initially high pro-tumor cell polarization, we used the M2 macrophage and Th2 cell concentrations predicted in the tumor microenvironment according to the mathematical model presented in [4], and the IL-4 and IL-10 concentrations used in [31].

### 2.3. Simulated Treatments

Supplementary Table S3 in Appendix B lists all the monotherapies that we considered, including a description of the dose, frequency and the time required for administration. These monotherapies served as the building blocks of the combination treatments that we simulated. ODEs were used to simulate the treatments as intravenous injections of constant infusion rate, or as capsules taken orally. The infusion rate terms that are listed were obtained by following the procedures described in [32] and we refer to them as the regular rates. Since M2 macrophages and Tregs are found in the tumor site at high concentrations, the rates of infusion of M1 macrophages and of Th1 helper cells were assigned the same value as the rate of infusion of NK cells. These M1 and Th1 infusion rates make it possible to significantly increase the M1-to-M2 macrophage ratio and the Th1-to-Th2 helper cell ratio. Doing so allowed us to quantify the effect of modulating these polarization ratios on treatment success.

As was done in [24], we assumed that wild-type cancer cells become resistant to Irinotecan chemotherapy in a dose-dependent manner. We also considered a hypothetical chemotherapy drug to which no cancer cells become resistant. Moreover, we assumed that in all simulations there were 35 KRAS-mutant cancer cells which are resistant to the monoclonal antibodies Panitumumab and Cetuximab. An objective of our project was to identify treatment combinations that are robust with respect to treatment resistance and that can eliminate the wild-type and the resistant cancer cells.

### 2.4. Model Parameters and Treatment Robustness

To investigate the effect of cytokine concentration on immune cell polarization and tumor growth, we used a partial differential equation (PDE) model of a granuloma that develops in response to a lung infection [31]. That model describes the immune reaction to an infection of *Mycobacterium tuberculosis*. It includes PDEs that simulate the spatiotemporal dynamics of pro- and anti-tumor alveolar macrophages, helper T cells, CTL and dendritic cells. The infection model also includes PDEs that describe the dynamics of the cytokines that are responsible for the polarization of alveolar macrophages and naïve helper T cells. The immune reaction against this bacterial infection is very similar to an immune reaction against cancer cells: the innate, adaptive and humoral immune responses are activated. Like the lung bacteria-immune system interaction, the same anti-tumor and pro-tumor cytokines play a role in the polarization of tumor-associated macrophages and helper T cells. A series of signaling pathways are turned on and off over time that lead to an initial inflammatory response characterized by a high concentration of M1 macrophages, Th1 cells and anti-tumor cytokines followed by an anti-inflammatory response characterized by a high concentration of M2 macrophages, Th2 cells and pro-tumor cytokines. Once the bacterial infection is resolved, the immune system returns to a state of homeostasis. In the case of cancer development, the immune response is unable to eliminate the tumor and becomes anergic. A high concentration of pro-tumor cells and cytokines remains in the tumor site, making the microenvironment highly immunosuppressive and the eradication of the tumor more difficult.

Supplementary Table S4 in Appendix B lists the parameter values that were used in the simulations. We incorporated into the ODE model [24] the cytokine-driven immune cell polarization and the antagonistic interactions of various cytokines simulated in the PDE infection model [31]. However, we assumed a homogeneous concentration of immune cells and cytokines in the tumor microenvironment. Hence, we used only the ordinary derivative terms of the PDE infection model. We also assumed that the rates of immune cell growth, polarization and cytokine production by polarized macrophages and helper T cells during a *Mycobacterium tuberculosis* infection are similar to those in a tumor-immune system interaction. The parameter values that we estimated were chosen based on similarities between two processes, or between cytokines that exert similar functions, as explained in [24]. The parameter values in [31] were converted to appropriate units for use in our model. For example, the units of concentration of the cytokines were converted from mg/L to IU/L by referring to the published specific activity of each cytokine. See [33] for details on how to perform these conversions. Cytokines whose specific activity was not available in the published literature were assigned an average specific activity of 1.3 × 10^10^ IU/g. Moreover, the density of macrophages, helper T cells and cytokines were reported in [31] in g/cm^3^. We converted the density of cells to cell/L and the density of cytokines to IU/L. The wet weight of an epithelial tumor having a volume of 1 cm^3^ is approximately 1 gram and contains approximately 10^8^ cancer cells [34]. Although different types of cells vary in size, we assumed a uniform sizefor cancer cells, macrophages and helper T cells and that there are 10^8^ cells per gram.

The parameters *d*, *1* and *s* together determine the level of strength of a patient’s immune response *D* by cytotoxic T lymphocytes (CTL), as defined by Equation (28). The 3 levels of immune response strength that we considered were the same as those defined in [32]. To better understand the effect of cytokine-driven pro-tumor cell polarization on treatment success, we considered nine cases. They are listed below in order from best-case scenario to worst-case scenario at the start of treatment:

1. Strong Immune Response: (*d* = 2.1,*l* =1.1,*s* = 5 × 10^−3^)
  - Best case: Low immunosuppression, low angiogenesis and no polarization.
  - High immunosuppression, high angiogenesis and no polarization.
  - High immunosuppression, high angiogenesis and high pro-tumor polarization.
2. Moderate Immune Response: (*d* =1.6,*l* =1.4,*s* = 8 × 10^−3^)
  - Low immunosuppression, low angiogenesis and no polarization.
  - High immunosuppression, high angiogenesis and no polarization.
  - High immunosuppression, high angiogenesis and high pro-tumor polarization.
3. Weak Immune Response: (*d* =1.3,*l* = 2,*s* = 4 × 10^−2^)
  - Low immunosuppression, low angiogenesis and no polarization.
  - High immunosuppression, high angiogenesis and no polarization.
  - Worst case: High immunosuppression, high angiogenesis and high pro-tumor polarization.

To ensure the safety in the clinic of all the simulated treatments, we followed the safety criteria described in [24]. In all the simulations, a complete response (CR) to a cancer treatment was defined as a tumor size at the end of the treatment period that is less than or equal to the diffusion-limited value of 1 × 10^6^ tumor cells. A partial response (PR) to treatment is described by a tumor that remains larger than 1 × 10^6^ cells, but that by the end of the treatment period is smaller than at the start of treatment. The no response (NR) classification applies when tumor size remains the same, or if the tumor becomes larger than it was at the start of treatment, by the time the treatment period ends. A cancer treatment that leads to a complete response at the three levels of immune response strength, for tumor sizes of up to 10^9^ cells, and that kills cancer cells that are resistant to Irinotecan and Panitumumab, was defined as being robust with respect to these perturbations. We defined a treatment that eliminated a tumor despite very low initial M1-to-M2 and Th1-to-Th2 ratios as being robust with respect to pro-tumor cell polarization.

## 3. Results

### 3.1. Strong Immune Response

**Figure 2.**
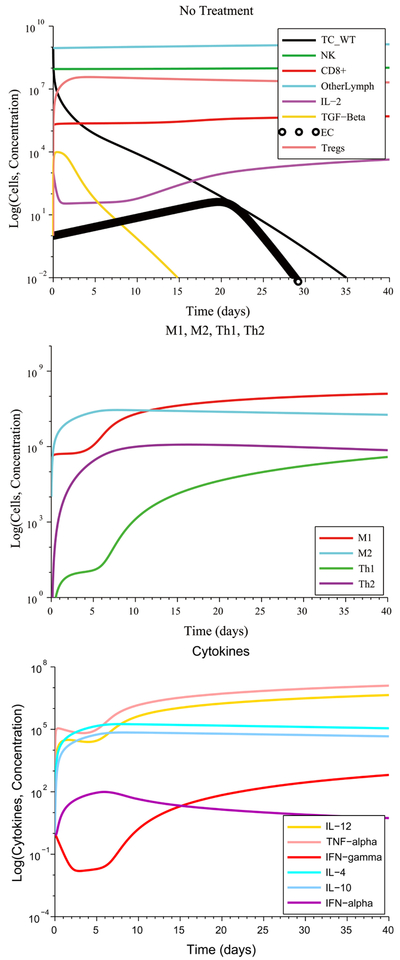
Without treatment, elimination of a tumor under a strong CTL response occurs whenever immunosuppression, tumor angiogenesis and immune cell polarization are low. The M1-to-M2 ratio and the Th1-to-Th2 ratio both increase, leading to an increased production of the anti-tumor cytokines IL-12, TNF-*α* and IFN-*γ* by M1 macrophages and Th1 helper cells.

We first considered the case of initially low immunosuppression, low angiogenesis and no initial pro-tumor cell polarization (refer to Supplementary Table S2 in Appendix B for the initial conditions that represent this scenario) coupled with a strong immune response. This scenario is not the typical one in the case of tumor growth. Tumors evade detection and destruction by reducing their antigenicity and by suppressing the immune system. Consequently, the immune response is attenuated and fails to eliminate the tumor. However, when we assumed the favorable conditions above, this led to the destruction of the tumor without the need for treatment, as can be seen in Figure 2. At steady-state, the M1-to-M2 macrophage polarization ratio is no more than one order of magnitude greater than 1, while the Th1-to-Th2 helper cell polarization ratio approaches 1. This result represents immune cell homeostasis after the cancer cells have been eliminated. The model predictions for this first scenario highlight the importance of reducing immunosuppression and tumor angiogenesis to allow the immune system to naturally eliminate the tumor.

**Figure 3.**
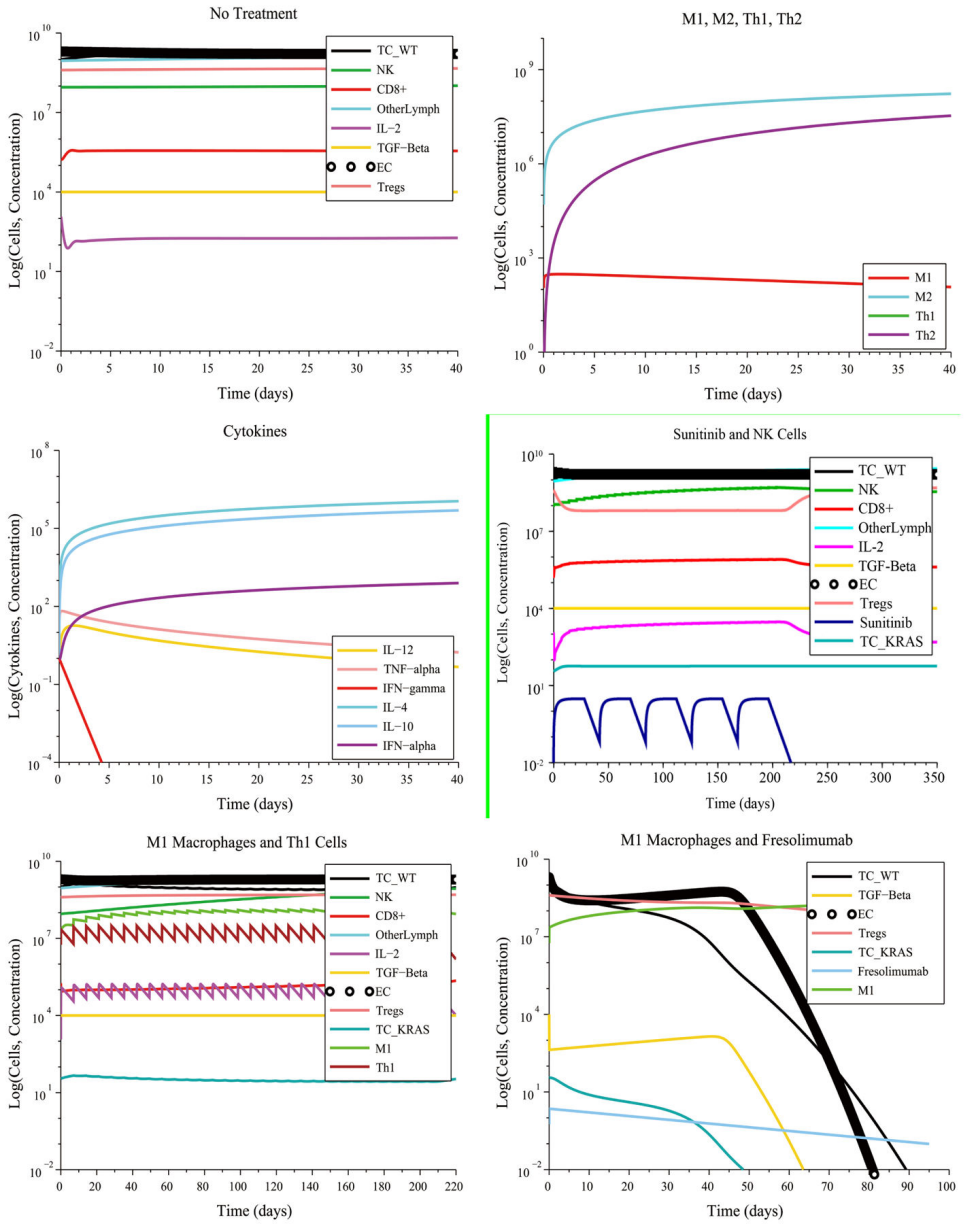
Initially high immunosuppression, angiogenesis and no cell polarization under a weak immune response leads to a low M1-to-M2 ratio and a low Th1-to-Th2 ratio without treatment. An M1 macrophage + Fresolimumab combination treatment eliminates the tumor in approximately 90 days by boosting the M1-to-M2 and Th1-to-Th2 ratios.

Figure 3 illustrates the case of initially high immunosuppression, high angiogenesis and no initial pro-tumor immune cell polarization. The immune system is unable to eliminate the tumor without treatment despite its ability to produce a strong CTL anti-tumor response. The M1-to-M2 and Th1-to-Th2 polarization ratios both remain close to zero due to the high initial concentration of M2 macrophages and of Th2 helper cells and throughout the simulation time. This occurs due to self-reinforcing positive feedback loops that lead to concentrations of pro-tumor cytokines that remain several orders of magnitude higher than the concentrations of the anti-tumor cytokines. The lopsided imbalance of these cytokine concentration perpetuates animmune cell polarization that favors tumor survival.

Under the initial conditions described above, the model predicts that a5-cycle Sunitinib + NK cell treatment given at their regular rates will fail to reduce the size of the tumor despite the significant reduction of the Treg population caused by the Sunitinib injections. This treatment fails due to the high concentration of immunosuppressive TGF-*β* at the start of the treatment and that remains high throughout the treatment period. Additionally, the model predicts that a Fresolimumab monotherapy consisting of 20 injections at the regular rate will also fail to eliminate the tumor. Similarly, 20 injections of M1 macrophages administered concurrently with 20 injections of Th1 cells at their regular rates is not sufficient to reduce the size of the tumor. An important result of this particular simulation is that injecting M1 macrophages and Th1 helper cells concurrently at their regular rates may not be sufficient to significantly alter the M1-to-M2 ratio and the Th1-to-Th2 ratio, if immunosuppression and angiogenesis are initially high. Other measures must be taken to modify these ratios, such as increasing the treatment dosage or disrupting additional signaling pathways. For example, one M1macrophage injection + one Fresolimumab (anti-TGF-*β*) injection administered concurrently at their regular rates eliminated the tumor in approximately 90 days. This success was due in part to the strong CTL response that the immune system is capable of exhibiting in this scenario, and the complementary nature at work of the two treatment modalities: a boosting of the CTL response coupled with a reduction of tumor angiogenesis and immunosuppression exerted by TGF-*β*.

**Figure 4.**
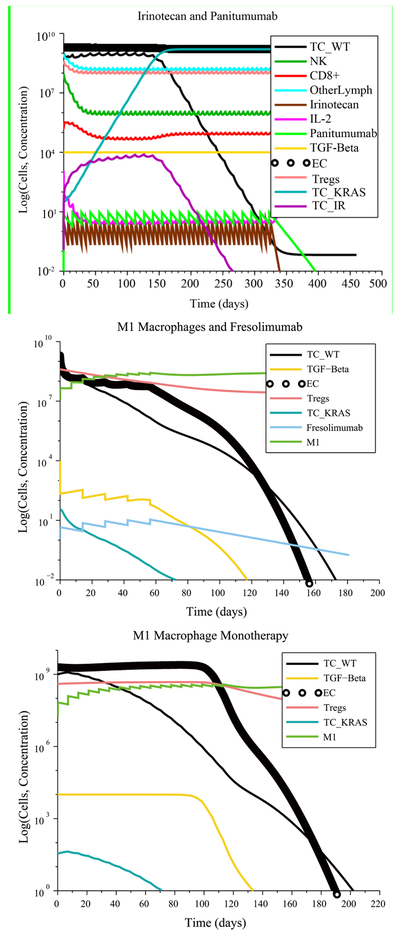
The KRAS-mutant cancer cells survive Panitimumab treatment but are eliminated by an M1 macrophage + Fresolimumab combination therapy in 172 days.

Figure 4 illustrates some unfavorable and favorable outcomes when assuming an initially high immunosuppression, high angiogenesis and high pro-tumor immune cell polarization. The combined administration of Irinotecan + Panitumumab eliminates some types of cancer cells, but not all. The wild-type cancer cells are killed by Irinotecan, by Panitumumab and by the immune system. The Irinotecan-resistant cells are eliminated by Panitumumab and by the immune system. However, over 50 Irinotecan injections and 25 Panitumumab injections must be administered, making this treatment impractical. Moreover, the Panitumumab-resistant cancer cells (KRAS-mutant) will survive. The result of this combination treatment will be a tumor consisting of Panitumumab-resistant cancer cells.

A more practical combination treatment that eliminates the tumor consists of giving 9M1 macrophage injections + 5 Fresolimumab injections both administered at 2 times their regular rate. The need for an increased dosage was due to the high pro-tumor immune cell polarization that existed at the start of treatment. The model predicts that a monotherapy consisting of17 M1 macrophage injections administered at three times the regular rate will also eliminate the tumor. These predictions highlight the importance of reducing tumor angiogenesis and immunosuppression as a treatment strategy. These results also suggest that a lack of reduction of tumor angiogenesis can be compensated by significantly increasing the M1-to-M2 macrophage polarization ratio by increasing the infusion rates of M1 macrophages into the tumor site. We surmise that a high M1-to-M2 polarization ratio leads to a rate of cancer cell destruction by immune cells that is greater than the rate of cancer cell replication, even when this replication rate is enhanced by angiogenesis.

### 3.2. Moderate Immune Response

**Figure 5.**
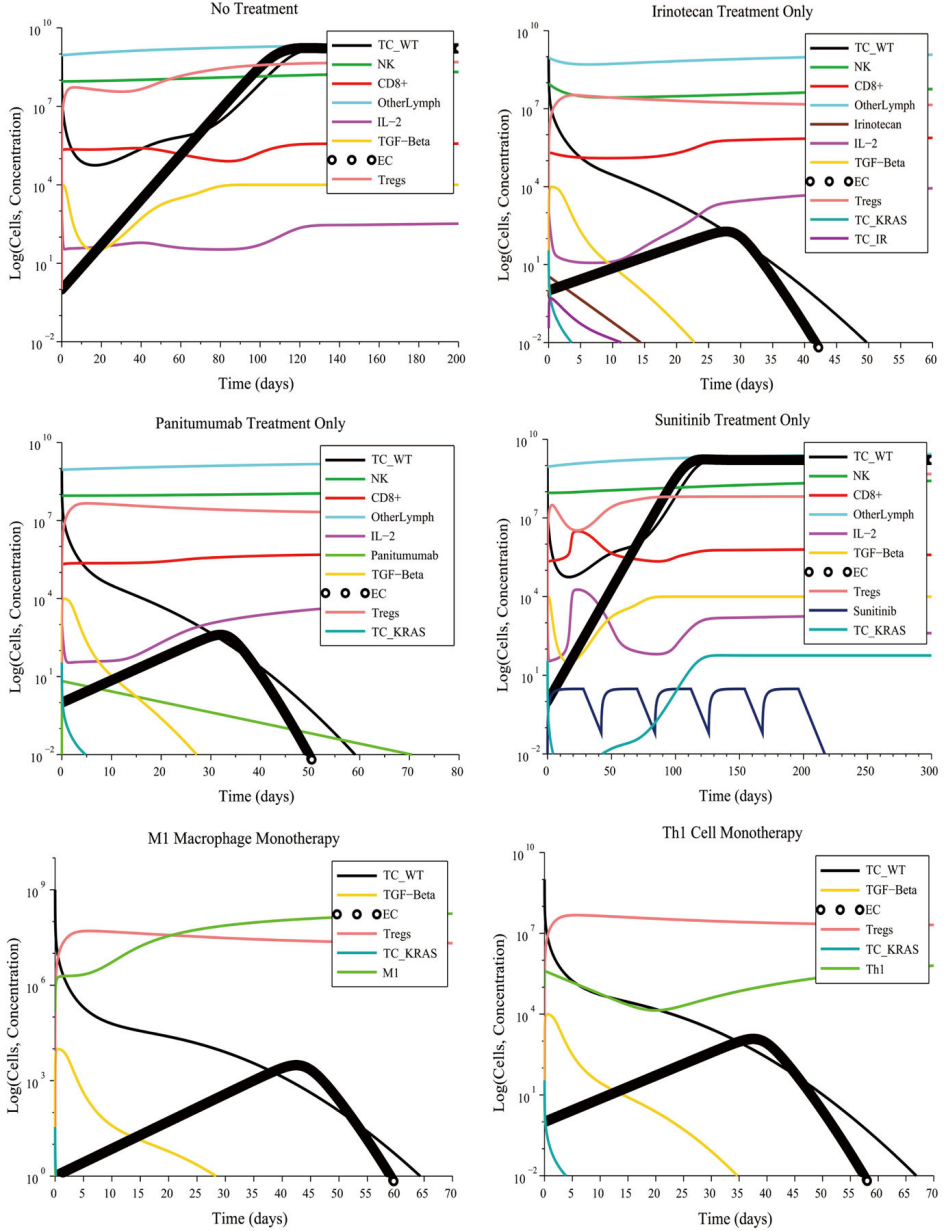
Tumor escape without treatment under a moderate response. Irinotecan monotherapy and Panitumumab monotherapy eliminate the wild-type and treatment-resistant cancer cells.

In the no treatment case with low immunosuppression, low angiogenesis and no initial immune cell polarization favorable to the tumor, the immune system initially decreases the size of the tumor. However, due to its moderate response, the immune system is unable to eliminate all the cancer cells and the tumor grows again to its maximum carrying capacity. With treatment, there were multiple treatment combinations that eliminated the tumor. A moderate immune response, without an initial pro-tumor immune cell polarization, made it possible for a single injection of Irinotecan or a single injection of Panitumumab administered at their regular rate to eliminate the tumor, including the Irinotecan-resistant and Panitumumab-resistant cells. This result is shown in Figure 5. An Irinotecan monotherapy can kill the wild-type and KRAS-mutant cells, while the Irinotecan-resistant cells are eliminated by the immune system. The moderate immune response with initially low immunosuppression, low angiogenesis and without initial pro-tumor polarization made it possible to eliminate the tumor by giving a single M1 macrophage or Th1 cell injection at a lower infusion rate (1 × 10^7^ cells L^−1^ day^−1^) than the regular rate (5.627 × 10^8^ cells L^−1^ day^−1^). However, a monotherapy consisting of 5 Sunitinib cycles administered at the regular rate had no effect on tumor growth. A reasonable explanation for this outcome is the fact that in this scenario, the Treg population was already low at the start of treatment and, hence, the Treg-targeted Sunitinib injections served no useful purpose.

**Figure 6.**
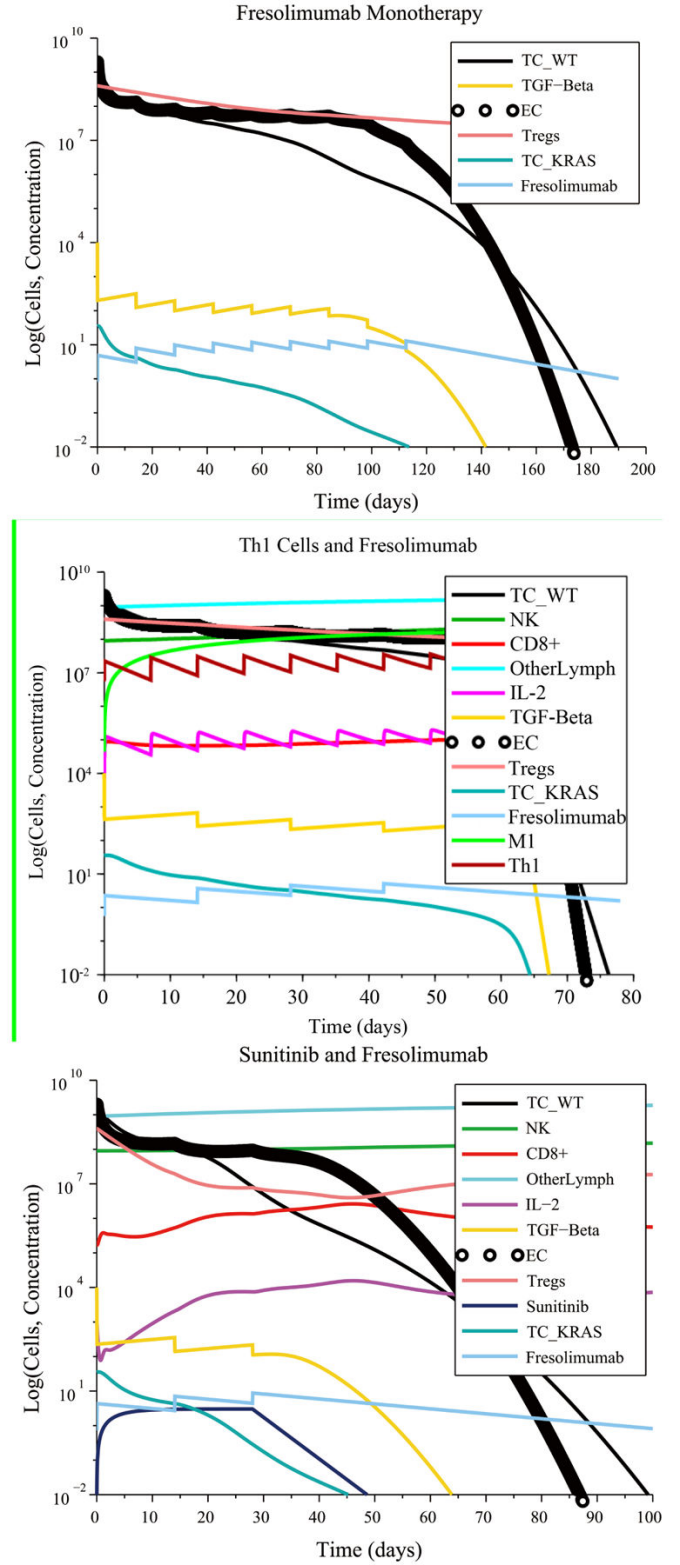
Combination treatments lead to a significantly shorter time to elimination of the tumor compared to Fresolimumab monotherapy.

In the case of high immunosuppression, high angiogenesis and no initial pro-tumor immune cell polarization, there were several combinations treatments that led to a complete response, as can be seen in Figure 6:

- 6 M1 macrophage injections + 3 Fresolimumab injections given at their regular rates
- 8 Th1 cell injections + 4 Fresolimumab injections given at their regular rates
- 1 cycle of Sunitinib administered at its regular ratein combination with 3 injections of Fresolimumab given at 1.28times its regular rate
- 9 Fresolimumab injections administered at 1.45 times the regular rate

Compared to Fresolimumab monotherapy, the combination treatments eliminated the tumor in a significantly shorter amount of time.

**Figure 7.**
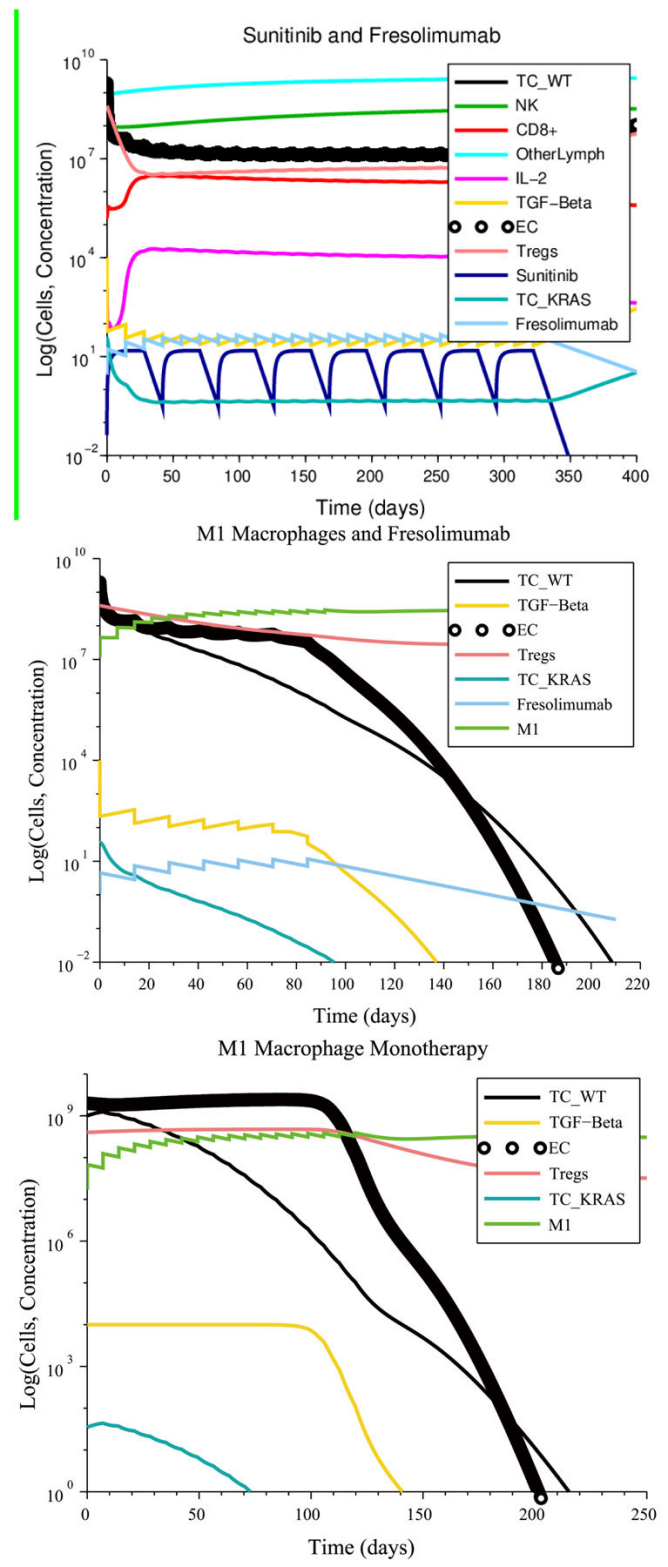
Pro-tumor cell polarization at the start of treatment requires increased doses of M1 Macrophages and Fresolimumab to eliminate a tumor.

Figure 7 shows the case of high immunosuppression, high angiogenesis and high pro-tumor immune cell polarization at the start of treatment. A Sunitinib + Fresolimumab treatment failed to eliminate the tumor even when administered at5 times their regular rates. A successful treatment consisted of administering14M1 macrophage injections together with 7 Fresolumumab injections, each given at two times their regular rate.Administering18M1 macrophage injections given at 3 times the regular rate also eliminated the tumor. The need for an increased dosage is due to the negative synergistic effect of high immunosuppression, angiogenesis and pro-tumor cell polarization at the start of treatment.

### 3.3. Weak Immune Response

**Figure 8.**
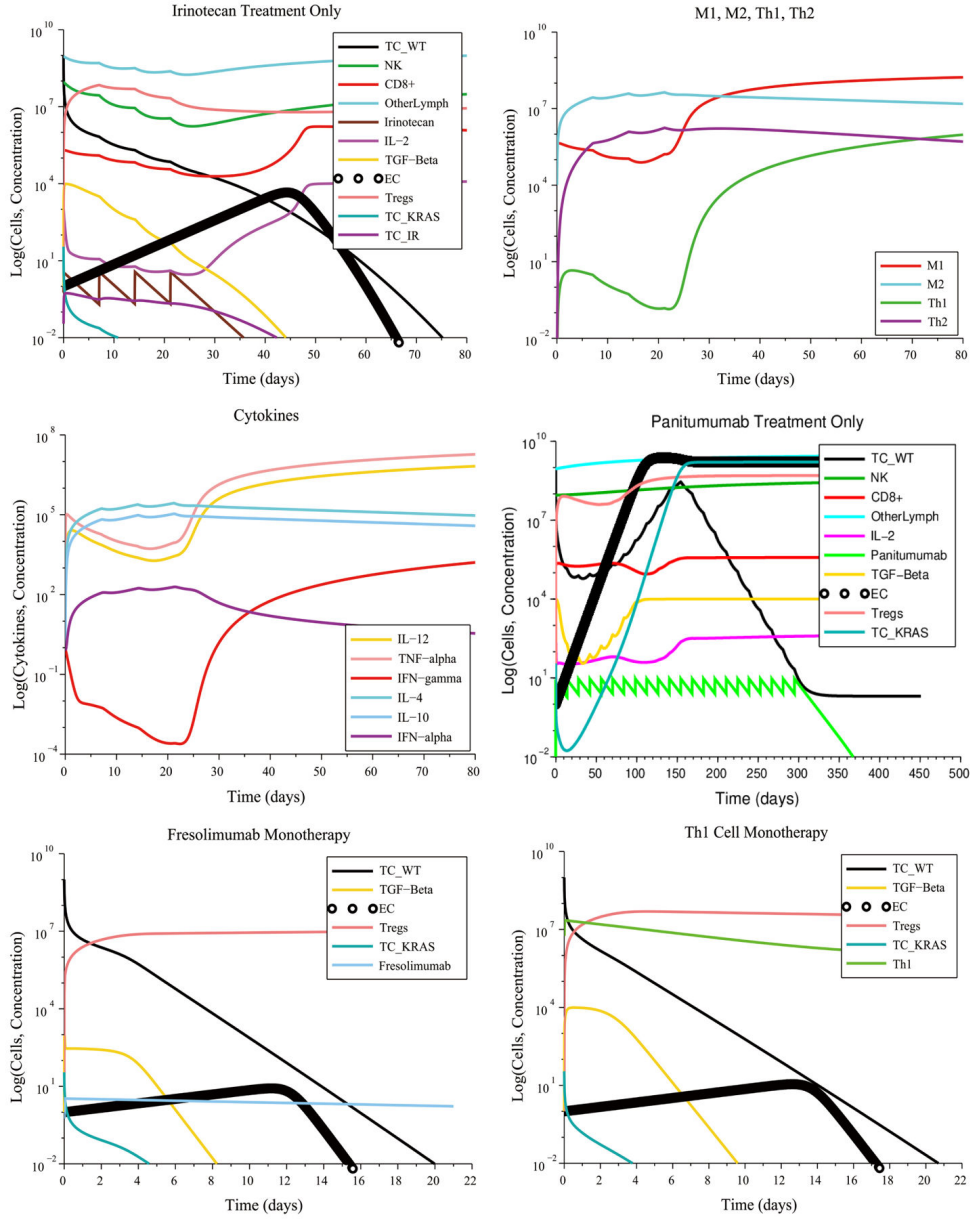
Irinotecan chemotherapy can eliminate the wild-type and mutant cancer cells if there are no pro-tumor polarized immune cells at the start of treatment, despite a weak CTL response. The cytokine plot shows that the concentration of pro-tumor cytokines IL-4, IL-10 and IFN-*α* released by M2 macrophages and Th2 helper cells decrease over time, leading to the success of Irinotecan chemotherapy.

In the case of low immunosuppression, low angiogenesis and no initial pro-tumor cell polarization (see Figure 8), an Irinotecan treatment at its regular rate eliminated the tumor with four injections. The chemotherapy-resistant cancer cells and the KRAS mutants were also eliminated due to a tumor microenvironment that was non-immunosuppressive, non-angiogenic and that contained no pro-tumor cells at the start of the treatment. As a result, the anti-tumor cytokines eventually increase in concentration, leading to M1-to-M2 and Th1-to-Th2 ratios that are representative of immune system homeostasis. In contrast, a treatment consisting of 22 Panitumumab injections, given at the regular rate, reduces the number of wild-type cancer cells, but the number of KRAS-mutant cells increases to the maximum carrying capacity, leading to treatment failure. Other successful treatments that eliminated all cancer cells consisted of a single injection of either Fresolimumab, M1 macrophages or Th1 helper cells given at their regular rate.

**Figure 9.**
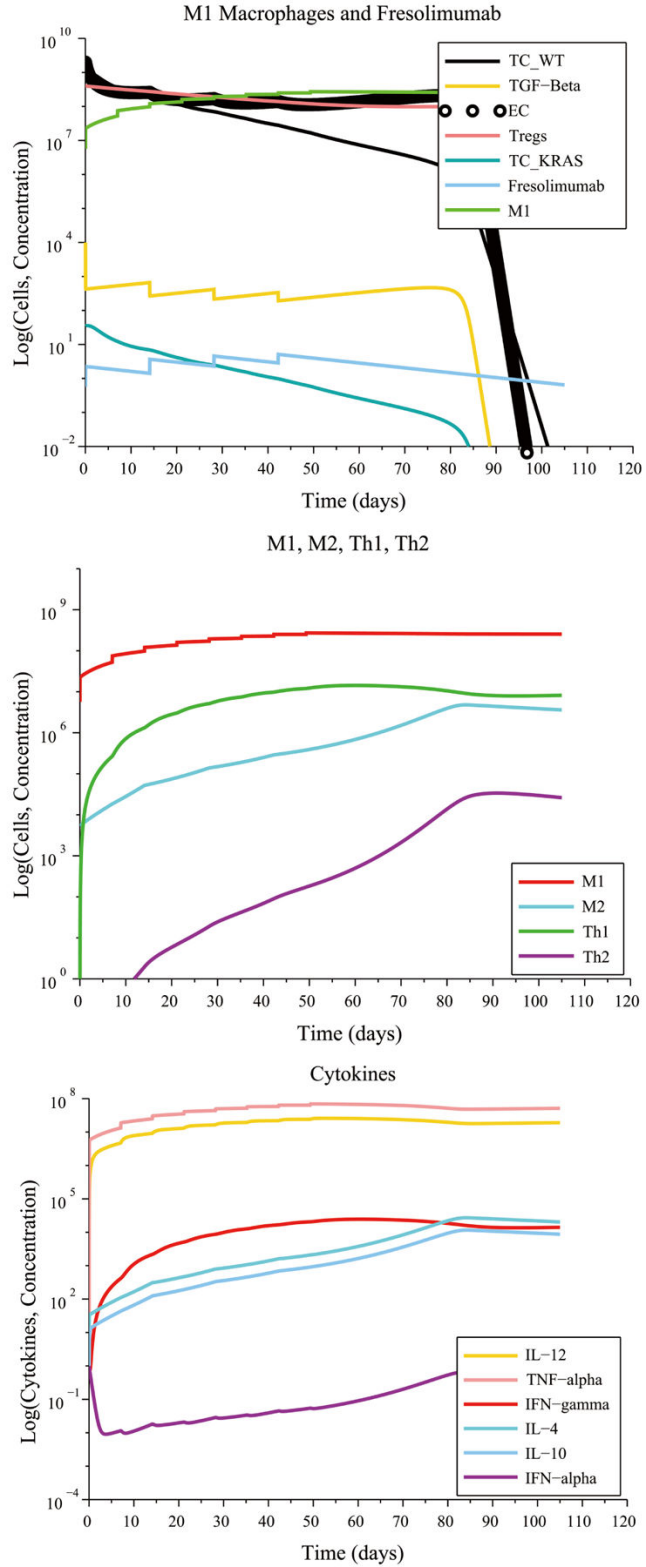
An M1 macrophage + Fresolimumab combination is an example of a treatment that is robust with respect to immune response strength, initial tumor size and treatment resistance.

In the case of high immunosuppression, high angiogenesis and no initial pro-tumor immune cell polarization, there were several combinations treatments that led to tumor elimination. The most notable case, shown in Figure 9, is the combination of 8 M1 macrophage injections coupled with 4 Fresolimumab injections, both given at their regular rates.

The M1 macrophage + Fresolimumab combination treatment was one of several treatments that were identified as being robust with respect to the level of immune response strength by CTL, with respect to the initial tumor size and with respect to resistance to Irinotecan and Panitumumab. These robust treatments, which are administered at their regular rates, are listed in Table 1.

In the worst-case scenario of high immunosuppression, high angiogenesis and high pro-tumor cell polarization with a weak immune response, there were no treatment combinations that could be administered at feasible rates that led to tumor elimination. Therefore, none of the treatments listed in Table 1 were found to be robust with respect to pro-tumor cell polarization. The failure of these promising treatments shows the negative impact on treatment success of the various positive feedback loops that reinforce pro-tumor immune cell polarization.

The lack of treatment robustness with respect to cell polarization, when assuming a weak immune response, motivated the authors to investigate the effect of directly disrupting the cytokine-driven feedback loops that are responsible for treatment failure. In general, when human cancers are diagnosed, they are well established and have already developed strong immunosuppressive mechanisms [35]. Therefore, we focused exclusively on the worst-case scenario of a weak immune response, high immunosuppression, angiogenesis, and cell polarization at the start of treatment. We noted that Fresolimumab reduces the concentration of free TGF-*β* in the tumor site. Therefore, Fresolimumab has the potential to reduce immunosuppression, tumor angiogenesis, and to some extent, to repo-larize M2 macrophages back to the M1 phenotype. However, IL-10 and IL-4 are also responsible for pro-tumor cell polarization. Administering anti-IL-10 monotherapy biweekly or in combination with cell-based treatments did not lead to a decrease in tumor size because IL-4 plays a role that is similar to that of IL-10. Therefore, we simulated a gene knockout experiment that reduces by 99% the production of IL-4 and IL-10by M2 macrophages and Th2 cells. We achieved this by decreasing the values of the parameters *λ*_*I*4*M*2_, *λ*_*I*4*T*2_, *λ*_*I*10*M*2_ and *λ*_*I*10*T*2_ by 99%. By doing so, the concentrations of IL-4 and IL-10 remained very low throughout the entire simulation time.

**Figure 10.**
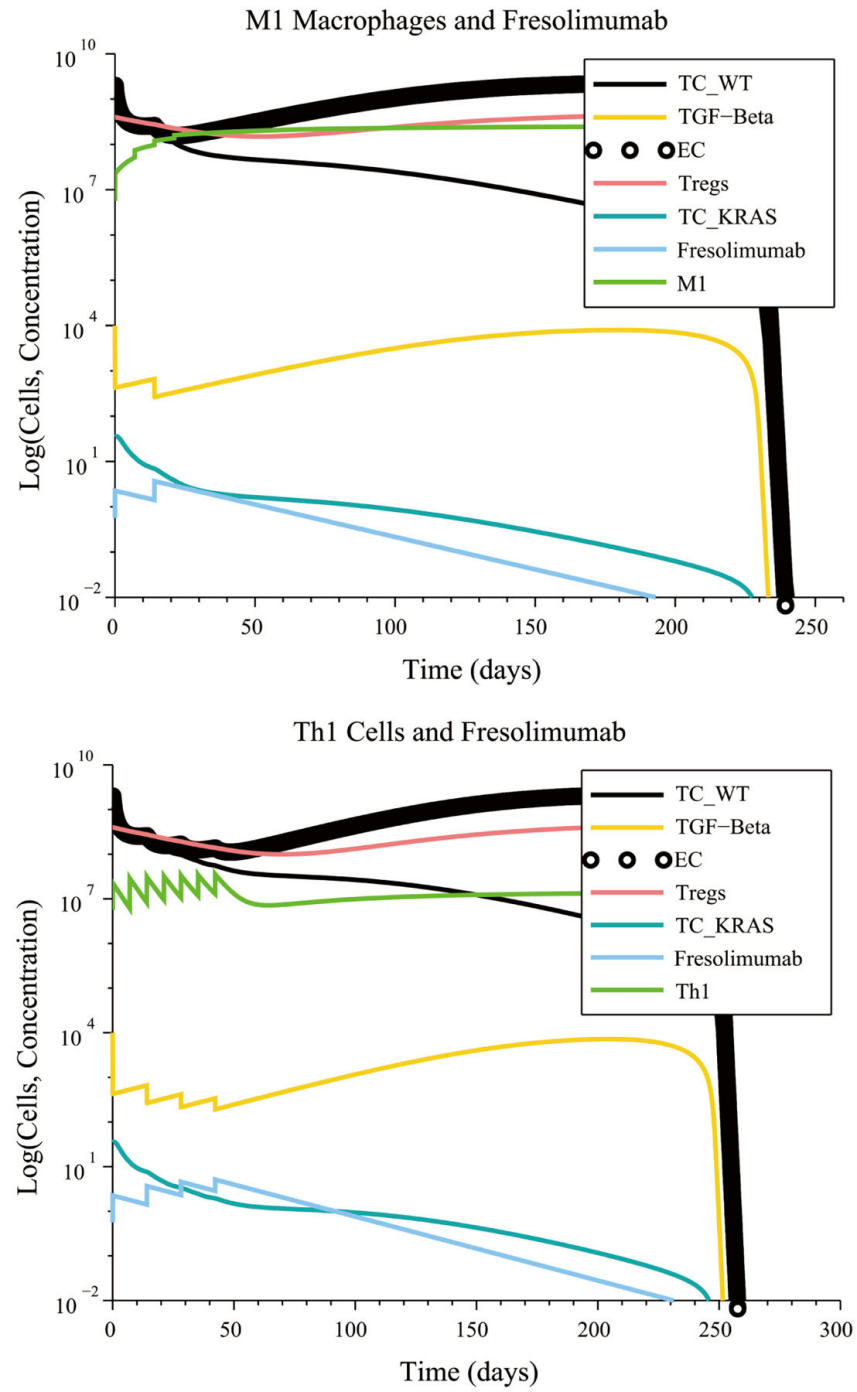
Reducing IL-4 and IL-10 production by 99% via a gene-knockout experiment makes an M1 macrophage + Fresolimumab treatment, as well as a Th1 helper cell + Fresolimumab treatment, robust with respect to pro-tumor cell polarization.

The gene knockout simulation showed that the tumor is eliminated when 4 M1 macrophage injections are administered together with 2Fresolimumab injections at their regular injection rates (see Figure 10). The tumor was also eliminated when 7 Th1 helper cell injections were administered together with 4Fresolimumab injections at their regular injection rates. These results show that neutralizing IL-4 and IL-10 should be part of an M1 macrophage + Fresolimumab or a Th1 cell + Fresolimumab treatment. This multi-pronged strategy confers these combination treatments with a robustness with respect to anti-tumor cell polarization, making them the most effective protocols that were identified.

### 3.4. Sensitivity Analysis

We proceeded to investigate the extent to which the predicted treatment outcomes depend on the values of the model parameters. To that end, we conducted a comprehensive sensitivity analysis of the weak immune response case with high immunosuppression, high angiogenesis and no cell polarization. The analysis consisted of decreasing (and increasing) each parameter value by 5% and keeping track of the percent change in the predicted tumor size at steady state. Table 2 lists some of the parameters that were varied and the resulting percent change in the tumor size. The results of this analysis indicated that the model is sensitive to changes to three parameters: *p*_1_ representing the maximum rate of TGF-*β* production by hypoxic tumor cells, *b*_*K*_ representing the proliferation rate of angiogenic endothelial cells, and *K*_*max*_ which is the rate at which TGF-*β* stimulates tumor growth. The sensitivity analysis indicates that the predictions of the model depend on the rate of tumor angiogenesis and growth. Therefore, we ensured that the pharmacokinetic and pharmacodynamical properties of TGF-*β* are simulated appropriately.

Of note is the fact that our model is significantly less sensitive to the production rate of IL-4 and IL-10 by M2 macrophages and by Th2 cells. This means that the predicted treatment outcomes are not affected by slight changes to the rate of production and decay of these cytokines.

## 4. Discussion

In this work, we focused on increasing the M1-to-M2 macrophage and the Th1-to-Th2 helper cell ratios by injecting anti-tumor polarized cells (M1 macrophages and Th1 helper cells) and by disrupting pro-tumor positive feedback loops driven by TGF-*β*, IL-4 and IL-10. An alternative approach to increase the M1-to-M2 macrophage and the Th1-to-Th2 helper cell ratios is to inject anti-tumor cytokines, such as IL-12 and IFN-*γ*, to reinforce the anti-tumor positive feedback loops that polarize macrophages and naïve helper T cells into the M1 and Th1 phenotypes, respectively. We did not include in our model an immunosuppressive positive feedback loop that involves the humoral immune system. It is known that Tregs and Bregs can reinforce each other’s pro-tumor phenotype through secretion of TGF-*β* and IL-10. We plan to include additional feedback loops into the model that involve the humoral immune response to assess their effect on treatment robustness. We will also consider additional sources of IL-10, such as Tregs and Bregs [36].

The cell-cell, cell-cytokine and cytokine-cytokine interactions were modeled according to previously published models by using first-order kinetics and by using Hill functions to account for rate saturation. Although there is not a prescribed way to simulate a given interaction, it is important to simulate an interaction in the most biologically-realistic manner. Therefore, it is worth checking whether the model predictions are sensitive to the approach taken when modeling certain interactions. In the future, we plan to undertake such an analysis by introducing Hill Functions of different orders to quantify their effect on treatment success.

In our model, we chose to make the maximum tumor size and tumor angiogenesis depend on TGF-*β* due to its multiple pro-tumor functions [37]. However, there are other cytokines that drive angiogenesis and that are also immunosuppressive, including the Vascular Endothelial Growth Factor (VEGF) [38] [39]. We will investigate whether the treatments listed in Table S4 remain robust when we let VEGF play the role of TGF-*β*. We will simulate injecting the monoclonal antibody bevacizumab (Avastin) to neutralize VEGF and will compare the effectiveness of an anti-VEGF treatment versus an anti-TGF-*β* treatment.

In the weak immune response case with high immunosuppression, high angiogenesis, and high pro-tumor cell polarization, it is possible to eliminate the tumor by administering a smaller number of Th1 helper cell injections. However, the drawback is that it would take longer to eliminate the tumor (over 300 days). This means that we would need to keep IL-4 and IL-10 at minimal concentration during this entire treatment period. This may not be advisable, especially if there are serious safety concerns of a prolonged reduced concentration of IL-4 and IL-10. For example, it has been shown that IL-10-deficient mice develop lethal uncontrolled inflammation of the intestine [40]. IL-10 stimulates the cytotoxicity of CTL [41] and a deficiency of IL-10 can lead to spontaneous tumor development [42]. Due to safety concerns, we plan to simulate concurrent injections of anti-IL-4 and anti-IL-10 instead of reducing the parameter values that represent IL-4 and IL-10 production by M2 macrophages and by Th2 cells. By doing so, we will be able to simulate a temporary and safer neutralization of IL-4 and IL-10, and will assess the effect of this cytokine blockade on treatment success.

Treatment effectiveness does not necessarily transfer from one cancer type to another. For example, it is quite possible that a treatment that can eliminate a slow-growing tumor will fail to stop the growth of a more aggressive tumor, such as glioblastoma. This aspect will be considered when we parametrize our model to identify the treatments that are most likely to eradicate a specific type of cancer. The purpose of our modeling project was primarily to assess the extent to which cytokine-driven feedback loops that perpetuate macrophage and helper T cell polarization into a pro-tumor phenotype determine treatment outcome. Second, we wanted to quantify the extent to which disrupting such feedback loops increases the likelihood of treatment success. We conducted a relative comparison between treatments of the time required for tumor elimination, the number of required injections and the required dose. The model results predicted that injecting M1 macrophages or Th1 cells, administering anti-TGF-*β* to reduce angiogenesis and simultaneously reducing the production of IL-4 and IL-10 is a promising strategy to eliminate a tumor.

## 5. Conclusion

Cytokine-driven feedback loops play an essential role in determining the steady-state dynamics of tumor-immune cell interactions, and the likelihood of treatment success. M1 macrophage + Fresolimumab and Th1 cell + Fresolimumab combination treatments were found to be robust with respect to the level of immune response strength, the initial tumor size and treatment resistance. The model predicts that treatments that simultaneously decrease tumor angiogenesis and boost the concentration of M1 macrophages or Th1 cells will be most effective, since they boost the M1-to-M2 and Th1-to-Th2 ratios. These treatments can be made robust with respect to pro-tumor immune cell polarization if coupled with antibodies that neutralize IL-4 and IL-10. Novel cancer treatments based on IL-10 and IL-4 antibodies could pave the way for tumor elimination despite a worst-case scenario at the start of treatment consisting of a highly immunosuppressive, angiogenic and polarized tumor microenvironment.

## Figures and Tables

**Table 1.** Treatments robust to immune strength level, tumor size and resistance.

Treatment Combinations	Treatment Description	Time to Elimination
M1 macrophages + Fresolimumab	8 weekly injections of M1 macrophages and 4 biweekly injections of F.	102 days
Th1 helper cells + Fresolimumab	17 weekly injections of Th1 helper cells and 9 biweekly injections of F.	160 days
M1 macrophages + Fresolimumab + Sunitinib	1 cycle of S concurrent with 3 biweekly injections of M1 and 3 biweekly injections of F.	56 days
Th1 helper cells + Fresolimumab + Sunitinib	2 cycles of S concurrent with 6 biweekly injections of Th1 and 6 biweekly injections of F (Th1 at a reduced dose of *v*_*T*1_ = 5 × 10^6^ cells/L · day).	108 days

**Table 2.** Sensitivity of the final tumor size to a 5% change to the parameter values.

Parameter	Description	% Change in Tumor Size	
		Parameter decreased by 5%	Parameter increased by 5%
*a*_*w*_	Growth rate of wild-type tumor cells.	−0.0001000%	0.0000905%
*T*_*K*_	Carrying capacity of wild-type and mutant tumor cells combined in the absence of tumor angiogenesis.	−0.0022626%	0.0022626%
*α*	Rate of circulating lymphocyte production.	0.0000786%	−0.0000782%
*p*_1_	Maximum rate of production of TGF-*β* by hypoxic tumor cells.	−1.8985642%	1.7851783%
*b*_1_	Critical tumor size at which the angiogenic switch occurs.	0.0000014%	−0.0000014%
*S*_1_	Concentration of TGF-*β* necessary to reduce the CD8+ T cell killing rate of tumor cells by half.	0.0000000%	−0.0000000%
*w*	Rate of Treg cell production.	−0.0000055%	0.0000054%
*b*_*k*_	Proliferation rate of angiogenic endothelial cells.	−2.0499774%	1.9076866%
*K*_max_	Rate at which TGF-*β* stimulates tumor growth.	−3.7233483%	3.6382439%
*e*	Rate of NK synthesis.	0.0000937%	−0.0000938%
*r*_1_	Rate of activation of CD8+ T cells due to NK cell-lysed tumor cell debris.	0.0000003%	−0.0000003%
*p*_*R*_	Rate of IL-2-induced Treg cell proliferation.	−0.0000097%	0.0000097%
*d*	Immune strength coefficient.	0.0000004%	−0.0000004%
*l*	Immune system strength scaling coefficient.	−0.0000097%	0.0000042%
*s*	Value describing how quickly CD8+ T cells respond to the presence of a tumor.	−0.0000004%	0.0000004%
*λ*_*Mo*_	Differentiation rate of monocytes to M1 macrophages and to M2 macrophages.	−0.0000004%	0.0000004%
$\lambda_{M_{2}}$	Maximal rate at which M1 macrophages are activated to become M2 macrophages.	−0.0000000%	0.0000000%
*M*_0_	Source term of monocytes.	−0.0000004%	0.0000004%
*T*_0_	Source term of naive helper T cells.	−0.0000721%	0.0000694%
$\lambda_{T_{2}}$	Production rate of Th2 cells.	−0.0000001%	0.0000001%
$\lambda_{I_{4}M_{2}}$	Production rate of IL-4 by M2 macrophages.	−0.0000000%	0.0000000%
$\lambda_{I_{10}M_{2}}$	Production rate of IL-10 by M2 macrophages.	−0.0000003%	0.0000002%
$\lambda_{I_{10}M_{2}}$	Production rate of IL-10 by Th2 cells.	−0.0000001%	0.0000001%

## Appendix A Model Equations

Equations (1)–(27) represent the ODEs of the mathematical model that we used to investigate the role of cytokine-driven polarization of immune cells on treatment success. Equation (28) was defined in [32] to set the level of strength of the anti-tumor immune response by cytotoxic T lymphocytes (CTL). It was expanded further by [24] and [25] to consider KRAS mutant and Irinotecan-resistant tumor cells and the immunosuppressive effect of TGF-*β* on the killing rate of tumor cells by CTL. We have expanded this term further to include the immunosuppressive effect of IL-10 on CTL. Equation (29) defines the cytokine-dependent polarization of monocytes and macrophages into an M1 phenotype and of naïve helper T cells into a Th1 phenotype. Similarly, Equation 30 defines the cytokine-dependent polarization of monocytes and macrophages into an M2 phenotype and of naïve helper T cells into a Th2 phenotype.

Wild-type Cancer Cells (sensitive to all the treatments):

(1)
$$\frac{\text{d}T_{w}}{\text{d}t}=a_{w}T_{w}\left(1-\frac{T_{w}+T_{cp}+T_{i}}{T_{K}+g_{2}E}\right)-\frac{\mu C_{1}}{K_{M}+C_{1}}T_{w}-\left(c+\xi \frac{A}{A+h_{1}}\right)\left({\text{e}}^{-\lambda_{T}R}\right)NT_{w}-DT_{w}\;-\left(K_{T}+K_{AT}A\right)\left(\frac{T_{w}}{T_{w}+\alpha_{1}T_{cp}}\right)\left(1-{\text{e}}^{-\delta_{T}\left(C_{1}+C_{2}\right)}\right)T_{w}-\Psi AT_{w}-\delta_{M1}M_{1}T_{w}-\delta_{T1}T_{1}T_{w}$$

KRAS-Mutant Cancer Cells (resistant to Panitumumab and Cetuximab):

(2)
$$\frac{\text{d}T_{cp}}{\text{d}t}=a_{m}T_{cp}\left(1-\frac{T_{w}+T_{cp}+T_{i}}{T_{K}+g_{2}E}\right)-\left(c+\xi \frac{A}{A+h_{1}}\right)\left({\text{e}}^{-\lambda_{T}R}\right)NT_{cp}-DT_{cp}\;-K_{T}\left(\frac{T_{w}}{T_{w}+\alpha_{1}T_{cp}}\right)\left(1-{\text{e}}^{-\delta_{TR}\left(C_{1}+C_{2}\right)}\right)T_{cp}-\delta_{M1}M_{1}T_{cp}-\delta_{T1}T_{1}T_{cp}$$

Chemotherapy-Resistant Cancer Cells (resistant to Irinotecan):

(3)
$$\frac{\text{d}T_{i}}{\text{d}t}=a_{m}T_{i}\left(1-\frac{T_{w}+T_{cp}+T_{i}}{T_{K}+g_{2}E}\right)+\frac{\mu C_{1}}{K_{M}+C_{1}}T_{w}-\left(c+\xi \frac{A}{A+h_{1}}\right)\left({\text{e}}^{-\lambda_{T}R}\right)NT_{i}-DT_{i}\;-\left(K_{T}+K_{AT}A\right)\left(\frac{T_{w}}{T_{w}+\alpha_{1}T_{cp}}\right)\left(1-{\text{e}}^{-\delta_{TR}C_{2}}\right)T_{i}-\psi AT_{i}-\delta_{M1}M_{1}T_{i}-\delta_{T1}T_{1}T_{i}$$

Natural Killer Cells:

(4)
$$\frac{\text{d}N}{\text{d}t}=eC-fN-\left(p+p_{A}\frac{A}{A+h_{1}}\right)N\left(T_{w}+T_{cp}+T_{i}\right)+\frac{p_{N}NI_{2}}{g_{N}+I_{2}}\;-K_{N}\left(1-{\text{e}}^{-\delta_{N}\left(C_{1}+C_{2}\right)}\right)N+v_{NK}$$

Cytotoxic T Lymphocytes:

(5)
$$\frac{\text{d}L}{\text{d}t}=-\frac{\theta mL}{\theta +I_{2}}+j\frac{T_{w}+T_{cp}+T_{i}}{k+T_{w}+T_{cp}+T_{i}}L-qL\left(T_{w}+T_{cp}+T_{i}\right)\;+\left(r_{1}N+r_{2}C\right)\left(T_{w}+T_{cp}+T_{i}\right)-\frac{uL^{2}RI_{2}}{\kappa +I_{2}}\;-K_{L}\left(1-{\text{e}}^{-\delta_{L}\left(C_{1}+C_{2}\right)}\right)L+\frac{p_{I_{2}}LI_{2}}{g_{I_{2}}+I_{2}}+v_{CTL}$$

Circulating T Lymphocytes in the Blood:

(6)
$$\frac{\text{d}C}{\text{d}t}=\alpha -\beta C-K_{C}\left(1-{\text{e}}^{-\delta_{C}\left(C_{1}+C_{2}\right)}\right)C$$

Activated Endothelial Cells:

(7)
$$\frac{\text{d}E}{\text{d}t}=b_{K}E\left(1-\frac{E}{\frac{K_{\text{max}}B}{g_{1}+B}}\right)-d_{K}E{\left(T_{w}+T_{cp}+T_{i}\right)}^{\frac{2}{3}}$$

Regulatory T Cells

(8)
$$\frac{\text{d}R}{\text{d}t}=wC-u_{R}R+\left(\frac{p_{R}RI_{2}}{g_{R}+I_{2}}\right)\left(\frac{B}{B+g_{\text{TGF}\;\beta }}\right)+\lambda_{\text{Treg}}T_{0}\left(\frac{B}{B+K_{\text{TGF}\;\beta }}\right)\;-h_{R}\left(1-{\text{e}}^{-\lambda_{R}S}\right)R-K_{R}\left(1-{\text{e}}^{-\delta_{R}\left(C_{1}+C_{2}\right)}\right)R$$

Chemotherapy Drug #1 (Irinotecan—Dose-dependent resistance emerges)

(9)
$$\frac{\text{d}C_{1}}{\text{d}t}=-\gamma_{1}C_{1}+v_{C1}$$

Chemotherapy Drug #2 (Hypothetical—No resistance emerges)

(10)
$$\frac{\text{d}C_{2}}{\text{d}t}=-\gamma_{2}C_{2}+v_{C2}$$

Interleukin-2:

(11)
$$\frac{\text{d}I_{2}}{\text{d}t}=-\mu_{I_{2}}I_{2}+\phi C+\frac{\omega LI_{2}}{\zeta +I_{2}}+\lambda_{I2T1}T_{1}+v_{I2}$$

Panitumumab and Cetuximab (monoclonal antibodies):

(12)
$$\frac{\text{d}A}{\text{d}t}=-\eta A-\lambda \left(T_{w}+T_{cp}+T_{i}\right)\left(\frac{A}{A+h_{2}}\right)+v_{A}$$

TGF-*β*:

(13)
$$\frac{\text{d}B}{\text{d}t}=p_{1}\frac{{\left(T_{w}+T_{cp}+T_{i}\right)}^{2}}{b_{1}^{2}+{\left(T_{w}+T_{cp}+T_{i}\right)}^{2}}-u_{1}B-b_{2}FB$$

Sunitinib (receptor tyrosine kinase inhibitor):

(14)
$$\frac{\text{d}S}{\text{d}t}=-\eta_{S}S+v_{S}$$

Fresolimumab (anti-TGF-*β*):

(15)
$$\frac{\text{d}F}{\text{d}t}=-\eta_{F}F-b_{3}BF+v_{F}$$

M1 Macrophages (anti-tumor):

(16)
$$\frac{\text{d}M_{1}}{\text{d}t}=\lambda_{M0}\frac{\varepsilon_{1}}{\varepsilon_{1}+\varepsilon_{2}}M_{0}+\lambda_{M1}\frac{\varepsilon_{1}}{\varepsilon_{1}+\varepsilon_{2}}M_{2}-\lambda_{M2}\frac{\varepsilon_{2}}{\varepsilon_{2}+\varepsilon_{1}}M_{1}\;\text{​}-K_{C}\left(1-{\text{e}}^{-\delta_{C}\left(C_{1}+C_{2}\right)}\right)M_{1}-d_{M1}M_{1}+v_{M1}$$

M2 Macrophages (pro-tumor):

(17)
$$\frac{\text{d}M_{2}}{\text{d}t}=\lambda_{M0}\frac{\varepsilon_{2}}{\varepsilon_{2}+\varepsilon_{1}}M_{0}-\lambda_{M1}\frac{\varepsilon_{1}}{\varepsilon_{1}+\varepsilon_{2}}M_{2}+\lambda_{M2}\frac{\varepsilon_{2}}{\varepsilon_{2}+\varepsilon_{1}}M_{1}\;-K_{C}\left(1-{\text{e}}^{-\delta_{C}\left(C_{1}+C_{2}\right)}\right)M_{2}-d_{M2}M_{2}$$

Th1 Helper T Cells (anti-tumor):

(18)
$$\frac{\text{d}T_{1}}{\text{d}t}=\lambda_{T1M1}T_{0}\frac{M_{1}}{M_{1}+K_{M1}}\frac{I_{12}}{I_{12}+K_{I12}}\frac{K_{I10}}{K_{I10}+I_{10}}+\lambda_{TI2}\frac{I_{2}}{I_{2}+K_{I2}}T_{1}\;-K_{C}\left(1-{\text{e}}^{-\delta_{C}\left(C_{1}+C_{2}\right)}\right)T_{1}-d_{T1}T_{1}+v_{T1}$$

Th2 Helper T Cells (pro-tumor):

(19)
$$\frac{\text{d}T_{2}}{\text{d}t}=\lambda_{T2}T_{0}\frac{M_{2}}{M_{2}+K_{M2}}\frac{I_{4}}{I_{4}+K_{I4}}\frac{K_{T1}}{K_{T1}+T_{1}}-K_{C}\left(1-{\text{e}}^{-\delta_{C}\left(C_{1}+C_{2}\right)}\right)T_{2}-d_{T2}T_{2}$$

Interleukin-1*β* (anti-tumor):

(20)
$$\frac{\text{d}I_{1\beta }}{\text{d}t}=\left(\lambda_{1\beta M_{1}}M_{1}+\lambda_{I1\beta T1}T_{1}\right)\left(\frac{K_{I\alpha }}{K_{I\alpha }+I_{\alpha }}\right)-d_{I1\beta }I_{1\beta }$$

Interleukin-4 (pro-tumor):

(21)
$$\frac{\text{d}I_{4}}{\text{d}t}=\left(\lambda_{I4M2}M_{2}+\lambda_{I4T2}T_{2}\right)\left(\frac{K_{I12}}{K_{I12}+I_{12}}\right)-d_{I4}I_{4}$$

Interleukin-10 (pro-tumor):

(22)
$$\frac{\text{d}I_{10}}{\text{d}t}=\lambda_{I10M2}M_{2}+\lambda_{I10T2}T_{2}-b_{4}A_{I10}I_{10}-d_{I10}I_{10}$$

Interleukin-12 (anti-tumor):

(23)
$$\frac{\text{d}I_{12}}{\text{d}t}=\frac{\lambda_{I12M1}M_{1}+\lambda_{I12T1}T_{1}}{\left(1+\frac{I_{4}}{K_{I4}}\right)\left(\frac{1+I_{10}}{K_{I10}}\right)}-d_{I12}I_{12}+v_{I12}$$

Tumor Necrotic Factor-*α* (anti-tumor):

(24)
$$\frac{\text{d}T_{\alpha }}{\text{d}t}=\frac{\lambda_{T\alpha M1}M_{1}+\lambda_{TaT1}T_{1}}{\left(1+\frac{I_{10}}{K_{I0}}\right)\left(\frac{1+I_{\alpha }}{K_{I\alpha }}\right)}-d_{T_{\alpha }}T_{\alpha }$$

Interferon-*α* (pro-tumor):

(25)
$$\frac{\text{d}I_{\alpha }}{\text{d}t}=\lambda_{I\alpha M_{2}}M_{2}\left(\frac{K_{I1\beta }}{K_{I1\beta }+I_{1\beta }}\right)-d_{I\alpha }I_{\alpha }$$

Interferon-*γ* (anti-tumor):

(26)
$$\frac{\text{d}I_{\gamma }}{\text{d}t}=\lambda_{I\gamma T1}T_{1}\frac{K_{I\alpha }}{K_{I\alpha }+I_{\alpha }}-d_{I\gamma }I_{\gamma }+v_{I\gamma }$$

Anti-Interleukin-10 (anti-tumor):

(27)
$$\frac{\text{d}A_{I10}}{\text{d}t}=-\eta_{AI10}A_{I10}-b_{5}I_{10}A_{I10}+v_{AI10}$$

Immune Response Strength by CTL:

(28)
$$D=\frac{d}{\left(1+\frac{B}{S_{1}}\right)\left(1+\frac{I_{10}}{T_{I10}}\right)}\cdot \frac{{\left(\frac{L}{T_{w}+T_{cp}+T_{i}}\right)}^{l}}{s+{\left(\frac{L}{T_{w}+T_{cp}+T_{i}}\right)}^{l}}$$

Polarization into Anti-Tumor Cells Driven by IFN-*γ* and TNF-*α*:

(29)
$$\varepsilon_{1}=\left(\lambda_{MI\gamma }\frac{I_{\gamma }}{I_{\gamma }+K_{I\gamma }}+\lambda_{MT\alpha }\frac{T_{\alpha }}{T_{\alpha }+K_{T\alpha }}\right)\left(\frac{K_{I10}}{K_{I10}+I_{10}}\right)\left(\frac{K_{\text{TGF}\beta }}{K_{\text{TGF}\beta }+B}\right)$$

Polarization into Pro-Tumor Cells Driven by IL-4 and IL-10:

(30)
$$\varepsilon_{2}=\left(\lambda_{MI4}\frac{I_{4}}{I_{4}+K_{I4}}+\lambda_{MI10}\frac{I_{10}}{I_{10}+K_{I10}}\right)\left(\frac{B}{B+K_{\text{TGF}\beta }}\right)$$

## Appendix B The Model Variables, Initial Conditions and Monotherapies

**Table S1.** The variables of the model.

Variable	Definition	Units
*T*_*w*_	Number of wild-type cancer cells that are sensitive to all treatments	cells
*T*_*cp*_	Number of mutant cancer cells resistant to cetuximab and panitumumab and that are sensitive to irinotecan and the hypothetical chemotherapy drug *C*_2_	cells
*T*_*i*_	Number of mutant cancer cells resistant to irinotecan and that are sensitive to cetuximab, panitumumab and the hypothetical chemotherapy drug *C*_2_	cells
*N*	Concentration of NK cells per liter of blood	cells/L
*L*	Concentration of CD8+ T cells per liter of blood	cells/L
*C*	Concentration per liter of blood of other circulating lymphocytes not including NK cells, CD8+ T cells or regulatory T cells	cells/L
*E*	Number of activated endothelial cells	cells
*R*	Concentration of regulatory T cells per liter of blood	cells/L
*C*_1_	Concentration of the chemotherapy agent Irinotecan per liter of blood to which cancer cells become resistant	mg/L
*C*_2_	Concentration of a hypothetical chemotherapy agent per liter of blood to which cancer cells do not develop resistance	mg/L
*I*_2_	Concentration of IL-2 per liter of blood	IU/L
*A*	Concentration of the monoclonal antibodies Cetuximab and Panitumumab per liter of blood	mg/L
*B*	Concentration of TGF-*β* per liter of blood	IU/L
*S*	Concentration of the tyrosine kinase inhibitor Sunitinib per liter of blood	mg/L
*F*	Concentration of the monoclonal antibody Fresolimumab per liter of blood	mg/L
*M*_1_	Concentration of M1 Macrophages per liter of blood	cells/L
*M*_2_	Concentration of M2 Macrophages per liter of blood	cells/L
*T*_1_	Concentration of Th1 cells per liter of blood	cells/L
*T*_2_	Concentration of Th2 cells per liter of blood	cells/L
*I*_1*β*_	Concentration of IL-1*β*per liter of blood	IU/L
*I*_4_	Concentration of IL-4 per liter of blood	IU/L
*I*_10_	Concentration of IL-10 per liter of blood	IU/L
*I*_12_	Concentration of IL-12 per liter of blood	IU/L
*T*_*α*_	Concentration of TNF-*α* per liter of blood	IU/L
*I*_*α*_	Concentration of IFN-*α* per liter of blood	IU/L
*I*_*γ*_	Concentration of IFN-*γ* per liter of blood	IU/L
*A*_*I*10_	Concentration of anti-IL-10 per liter of blood	mg/L

**Table S2.** The initial conditions of the model.

Variable	Units	Scenario 1 LI, LA, NP	Scenario 2 HI, HA, NP	Scenario 3 HI, HA, HP
^*T*^ *w*	cells	1 × 10^9^	1 × 10^9^	1 × 10^9^
*T*_*cp*_	cells	35	35	35
*T*_*i*_	cells	0	0	0
*N*	cells/L	9 × 10^7^	9 × 10^7^	9 × 10^7^
*L*	cells/L	1.8 × 10^5^	1.8 × 10^5^	1.8 × 10^5^
*C*	cells/L	9 × 10^8^	9 × 10^8^	9 × 10^8^
*E*	cells	1	2 × 10^9^	2 × 10^9^
*R*	cells/L	1	4 × 10^8^	4 × 10^8^
*C*_1_	mg/L	0	0	0
*C*_2_	mg/L	0	0	0
*I*_2_	IU/L	1173	1173	1173
*A*	mg/L	0	0	0
*B*	IU/L	1	1 × 10^4^	1 × 10^4^
*S*	mg/L	0	0	0
*F*	mg/L	0	0	0
*M*_1_	cells/L	0	0	0
*M*_2_	cells/L	0	0	1 × 10^5^
*T*_1_	cells/L	0	0	0
*T*_2_	cells/L	0	0	9 × 10^7^
*I*_1*β*_	IU/L	1	1	1
*I*_4_	IU/L	1	1	2.21 × 10^5^
*I*_10_	IU/L	1	1	2.235 × 10^3^
*I*_12_	IU/L	1	1	1
*T*_*α*_	IU/L	1	1	1
*I*_*α*_	IU/L	1	1	1
*I*_*γ*_	IU/L	1	1	1
*A*_*I*10_	mg/L	0	0	0

**Table S3.** Monotherapy dose and frequency.

Treatment	Agent Type	Dose and Frequency	Infusion Time	Infusion Rate
Chemotherapy	Irinotecan [32]	125 mg/m^2^ given weekly. This cycle may be repeated.	1.5 hr	*v*_*C*1_ = 57.947 mg/L · day
Chemotherapy	(Hypothetical)	125 mg/m^2^ given weekly. This chemotherapy agent was assumed to have the same cytotoxic properties on the tumor and immune cells as Irinotecan. It was assumed that tumor cells never develop resistance to this drug. This cycle may be repeated.	1.5 hr	*v*_*C*2_ = 57.947 mg/L · day
Monoclonal antibody	Cetuximab [32]	Loading dose (LD): 400 mg/m^2^ (a one-time injection before giving the maintenance doses) Maintenance dose (MD): 250 mg/m^2^ (given weekly – start one week after giving LD and rest 2 weeks every 4 weeks). This cycle may be repeated.	LD: 2 hr MD: 1 hr	LD: *v*_*A*_ = 139.072mg/L · day MD: *v*_*A*_ = 173.840 mg/L · day Note: the same infusion term *v*_*A*_ is used for cetuximab and for panitumumab, but their *v*_*A*_ values are different.
Monoclonal antibody	Panitumumab [32]	6 mg/kg every two weeks. No loading dose is required. This cycle may be repeated.	1 hr	*v*_*A*_ = 168.816 mg/L · day
Monoclonal antibody	Fresolimumab (anti-TGF-β) [43]	3 mg/kg every two weeks. This cycle may be repeated.	1.5 hr	*v*_*F*_ = 56.272 mg/L · day
Monoclonal antibody	Anti-IL-10 Est. from [44]	3 mg/kg every two weeks. This cycle may be repeated.	1.5 hr	*v*_*AI*10_ = 21.0 mg L · day
RTK inhibitor	Sunitinib (anti-Treg) [45]	A Sunitinib capsule is given daily for 28 straight days followed by two weeks of rest. In total, 23.447 mg of sunitinib are administered per each 6-week cycle. This cycle may be repeated.	None	*v*_*s*_ = 0.8374 mg/L · day
Adoptive cell transfer	NK cells [46]	An intravenous injection of 2 × 10^7^ NK cells per kg is given weekly. This cycle may be repeated.	1 hr	*v*_*NK*_ = 5.627 × 10^8^ cells/L · day
Adoptive cell transfer	CTL [47]	Five intravenous injections of 1 × 10^10^ CTL per m^2^ are given every 5 days and they are followed by 45 days of rest. This 65-day cycle may be repeated.	1 hr	*v*_*CTL*_ = 6.9536 × 10^9^ cells/L · day
Cytokine Treatment	IL-2 [48]	180,000 IU/kg injected over 15 minutes every 8 hours.	0.25 hr	*v*_*IL*2_ = 2.0258 × 10^7^ IU/L · day
Adoptive cell transfer	M1 macrophages	An intravenous injection of 2 × 10^7^ M1 macrophages per kg is given weekly. This cycle may be repeated.	1 hr	*v*_*M*1_ = 5.627 × 10^8^ cells/L · day
Adoptive cell transfer	Th1 helper cells	An intravenous injection of 2 × 10^7^ Th1 cells per kg is given weekly. This cycle may be repeated.	1 hr	*v*_*T*1_ = 5.627 × 10^8^ cells/L · day

**Table S4.** The parameters of the model.

Parameter	Description	Value	Units	Ref.
*a*_*w*_	Tumor growth rate (colorectal cancer)	2.31 × 10^−1^	day^−1^	[32]
*c*	Rate of NK cell-induced tumor death	5.156 × 10^−14^	L cell^−1^ day^−1^	[32]
*d*	Immune strength coefficient	{1.3, 1.6, 2.1}	day^−1^	[32]
*1*	Immune system strength scaling coefficient	{1.1, 1.4, 2}	unitless	[32]
*s*	Value describing how quickly CD8+ T cells respond to the presence of a tumor	{5×10^−3^, 8×10^−3^, 4×10^−2^}	L	[32]
*ξ*	Rate of NK cell-induced tumor death through antibody-dependent cellular cytotoxicity (ADCC)	6.5 × 10^−6^ (0 for panitumumab)	L cell^−1^ day^−1^	[32]
*h*_1_	Concentration of mAbs necessary for half-maximal increase in ADCC activity	1.25 × 10^−6^ (0 for panitumumab)	mg L^−1^	[32]
*X*	Determines the percent level of the maximum rate of chemotherapy-induced tumor death of wild-type and mutant cells	0.75	unitless	[32]
*K*_*T*_	Death rate of wild-type and mutant tumor cells due to chemotherapy	(8.1 × 10^−1^)· X	day^−1^	[25], [32]
*K*_*AT*_	Chemotherapy-induced death rate of wild-type and mutant tumors due to mAbs cetuximab and panitumumab	4 × 10^−4^	L mg^−1^ day^−1^	[32]
*δ*_*T*_	Chemotherapy efficacy coefficient on wild-type and mutant cancer cells	2 × 10^−1^	L mg^−1^	[32]
*Y*	Determines the percent level of the maximum rate of mAb-induced tumor death of wild-type and mutant cells	0.75	unitless	[32]
*ψ*	Rate of mAb-induced wild-type and mutant tumor cell death	(2.28 × 10^−2^)· *Y* for cetuximab (3.125 × 10^−2^)· Y for panitumumab	L mg^−1^ day^−1^	[32]
*f*	Rate of NK cell turnover	1 × 10^−2^	day^−1^	[32]
*e*	Rate of NK synthesis from circulating lymphocytes	$\frac{1}{9}\cdot f$	day^−1^	[32]
*g*_*N*_	IL-2 concentration needed for half-maximal NK cell proliferation	2.5036 × 10^5^	IU L^−1^	[32]
*p*_*N*_	Rate of IL-2-induced NK cell proliferation	5.13 × 10^−2^	day^−1^	[32]
*p*	Rate of NK cell death due to interaction with the tumor	5.156 × 10^−14^	cell^−1^ day^−1^	[32]
*p*_*A*_	Rate of NK cell death due to interaction with mAbs complexes	6.5 × 10^−10^ (0 for panitumumab)	cell^−1^ day^−1^	[32]
*K*_*N*_	Rate of NK cell depletion due to chemotherapy toxicity	9.048 × 10^−1^	day^−1^	[32]
*δ*_*N*_	Coefficient of chemotherapy toxicity on NK cells	2 × 10^−1^	L mg^−1^	[32]
*m*	Rate of turnover of activated CD8+ T cells	5 × 10^−3^	day^−1^	[32]
*θ*	IL-2 concentration required to halve the CD8+ T cell turnover rate	2.5036 × 10^−3^	IU L^−1^	[32]
*q*	Rate of CD8+ T cell death due to interaction with the tumor	5.156 × 10^−17^	cell^−1^ day^−1^	[32]
*r*_1_	Rate of activation of CD8+ T cell due to NK cell-lysed tumor cell debris	5.156 × 10^−12^	cell^−1^ day^−1^	[32]
*r*_2_	Rate of CD8+ T cell production from circulating lymphocytes	1 × 10^−15^	cell^−1^ day^−1^	[32]
*p*_*I*2_	Rate of CD8+ T cell activation induced by IL-2	2.4036	day^−1^	[32]
*g*_*I*2_	Concentration of IL-2 necessary for half-maximal CD8+ T cell activation	2.5036 × 10^3^	IU L^−1^	[32]
*u*	Rate of inhibition of surplus CD8+ T cells induced by Treg cells in the presence of IL-2	2.3085 × 10^−13^	L^2^ cell^−2^ day^−1^	[49]
*κ*	Concentration of IL-2 required to halve the immunosuppressive effect of Treg cells on CD8+ T cells	2.5036 × 10^3^	IU L^−1^	[49]
^*j*^	Rate of activation of CD8+ T cells due to CD8+ T cell-lysed tumor cell debris	1.245 × 10^−4^	day^−1^	[32]
*k*	Tumor size required for half-maximal CD8+ T cell activation by CD8+ T cell-lysed tumor cell debris	2.019 × 10^7^	cells	[32]
*K*_*L*_	Rate of CD8+ T cell depletion from chemotherapy toxicity	4.524 × 10^−1^	day^−1^	[32]
*δ*_*L*_	Coefficient of chemotherapy toxicity on CD8+ T cells	2 × 10^−1^	L mg^−1^	[32]
*β*	Rate of circulating lymphocyte turnover	6.3 × 10^−3^	day^−1^	[32]
*α*	Rate of circulating lymphocyte production	(3 × 10^9^)·*β*	cells L^−1^ day^−1^	[32]
*K*_*C*_	Rate of lymphocyte depletion from chemotherapy toxicity	5.7 × 10^−1^	day^−1^	[32]
*δ*_*C*_	Coefficient of chemotherapy toxicity on circulating lymphocytes	2 × 10^−1^	L mg^−1^	[32]
*μ*_*I*2_	Rate of excretion and elimination of IL-2	11.7427	day^−1^	[32]
*ω*	Rate of IL-2 production from CD8+ T cells	7.88 × 10^−2^	IU cell^−1^ day^−1^	[32]
*ϕ*	Rate of IL-2 production from circulating CD4+ and naive CD8+ T cells	1.788 × 10^−7^	IU cell^−1^ day^−1^	[32]
*ζ*	Concentration of IL-2 for half-maximal CD8+ T cell IL-2 production	2.5036 × 10^3^	IU L^−1^	[32]
*γ*_1_	The rate of excretion and elimination of irinotecan	4.077 × 10^−1^	day^−1^	[32]
*η*	Rate of cetuximab and panitumumab turnover and excretion	1.386 × 10^−1^ for cetuximab 9.242 × 10^−2^ for panitumumab	day^−1^	[32]
*λ*	Rate of mAb-tumor cell complex formation	8.9 × 10^−14^ for cetuximab 8.6 × 10^−14^ for panitumumab	mg cell^−1^ L^−1^ day^−1^	[32]
*h*_2_	Concentration of cetuximab or panitumumab for half-maximal EGFR binding	4.45 × 10^−5^ for cetuximab 4.3 × 10^−5^ for panitumumab	mg L^−1^	[32]
*T*_*K*_	Carrying capacity of wild-type and mutant tumor cells combined in the absence of tumor angiogenesis	1 × 10^6^	cells	[50]
*g*_2_	Conversion factor from number of activated endothelial cells to the increase in tumor carrying capacity	1	$\frac{\text{tumor cells}}{\text{endothelial cell}}$	Est.
*p*_1_	Maximum rate of production of TGF-β by hypoxic tumor cells	1 × 10^5^	IU L^−1^ day^−1^	Est. from [50]
*b*_1_	Critical tumor size at which the angiogenic switch occurs	1 × 10^6^	cells	[50]
*S*_1_	Concentration of TGF-*β* necessary to reduce the CD8+ T cell killing rate of tumor cells by half	7 × 10^4^	IU L^−1^	[51]
*u*_1_	Decay rate of TGF-*β*	10	day^−1^	[50]
*b*_*K*_	Proliferation rate of angiogenic endothelial cells	0.198	day^−1^	[52]
*K*_max_	Maximum carrying capacity for blood vessel growth stimulated by TGF-*β* secreted by tumor cells	5 × 10^9^	cells	Est.
*g*_1_	TGF-*β* concentration that gives a half-maximal proliferation rate of endothelial cells	1 × 10^4^	IU L^−1^	Est.
*d*_*K*_	Growth inhibition coefficient of endothelial cells by tumor cells	5 × 10^−8^	cell^−2/3^ day^−1^	Est. from [53]
*λ*_*T*_	Suppressive effect of Treg cells on wild-type and mutant tumor cell kill rate by NK cells	1.59 × 10^−9^	L cell^−1^	[45]
*w*	Rate of Treg cell production from circulating lymphocytes	4.698 × 10^−4^	day^−1^	[45]
*u*_*R*_	Rate of Treg cell turnover	3.851 × 10^−2^	day^−1^	[54]
*p*_*R*_	Rate of IL-2-induced Treg cell proliferation	3.598 × 10^−2^	day^−1^	[49]
*g*_*R*_	Concentration of IL-2 necessary for half-maximal activation of Treg cells	11.027	IU L^−1^	[49]
*h*_*R*_	Rate of Treg cell inhibition by Sunitinib	0.227	day^−1^	[45]
*K*_*R*_	Rate of Treg cell depletion from chemotherapy toxicity	5.7 × 10^−1^	day^−1^	[45]
*α*_1_	Determines the scope of influence of KRAS-mutant tumor cells *T*_*cp*_ in making tumor cells resistant to chemotherapy and in reducing the sensitizing role of Cetuximab and Panitumumab to chemotherapy	1 × 10^7^	unitless	[25]
*λ*_*R*_	Efficacy of Sunitinib in inhibiting the immunosuppressive activity of Tregs	50.02	L mg^−1^	[45]
*δ*_*R*_	Chemotherapy toxicity on Tregs	2 × 10^−1^	L mg^−1^	[32]
*η*_*s*_	Rate of excretion and elimination of Sunitinib	0.277	day^−1^	[45]
*a*_*m*_	Growth rate of mutant tumor cells (colorectal cancer)	2.31 × 10^−1^	day^−1^	[32]
*μ*	Maximum mutation rate of wild-type tumor cells	4 × 10^−5^	day^−1^	[55]
*K*_*M*_	Concentration of Irinotecan chemotherapy that leads to a half-maximal rate of mutation of wild-type tumor cells *T*_*w*_ into irinotecan-resistant tumor cells *T*_*i*_	1 × 10^3^	mg L^−1^	Est.
*δ*_*TR*_	Efficacy of the second type of chemotherapy drug of killing irinotecan-resistant tumor cells	2 × 10^−1^	L mg^−1^	[32]
*γ*_2_	Rate of excretion and elimination of the hypothetical chemotherapy drug	4.077 × 10^−1^	day^−1^	[32]
*η*_*F*_	Degradation rate of anti-TGF-beta (Fresolimumab)	0.033	day^−1^	Est. from [56]
*b*_2_	Rate of loss of free TGF-*β* due to binding with anti-TGF-*β*	100	L mg^−1^ day^−1^	Est. from [56]
*b*_3_	Rate of loss of free anti-TGF-*β* due to binding with TGF-*β*	2.5 × 10^−13^	L IU^−1^ day^−1^	Est. from [56]
*λ*_*M* 0_	Differentiation rate of M0 macrophages into an M1 or M2 macrophage	1 × 10^−4^	day^−1^	Est. from [31]
*λ*_*M* 1_	Maximal rate at which M2 macrophages switch phenotype to become M1 macrophages	6 × 10^−3^	day^−1^	[31]
*λ*_*M* 2_	Maximal rate at which M1 macrophages switch phenotype to become M2 macrophages	6 × 10^−3^	day^−1^	Est. from [31]
*λ*_*MI* 4_	Production rate of M2 macrophages due to IL-4	1 × 10^−3^	day^−1^	[31]
*λ*_*MIγ*_	Production rate of M1 macrophages due to IFN-*γ*	1 × 10^−3^	day^−1^	[31]
*λ*_*MIα*_	Production rate of M1 macrophages due to TNF-*α*	1 × 10^−3^	day^−1^	[31]
*λ*_*T*1*M*1_	Production rate of Th1 cells by M1 macrophages and IL-12	0.23	day^−1^	[31]
*λ*_*TI*2_	Production rate of Th1 cells by IL-2	1	day^−1^	[31]
*λ*_*T*2_	Production rate of Th2 cells	0.8	day^−1^	[31]
*λ*_*IγT*1_	Production rate of IFN-*γ* by Th1 cells	3.731 × 10^−3^	IU cell^−1^ day^−1^	[31]
*λ*_*I*12*M*1_	Production rate of IL-12 by M1 macrophages	0.13	IU cell^−1^ day^−1^	Est. from [31]
*λ*_*I*2*T*1_	Production rate of IL-2 by Th1 cells	0.0672	IU cell^−1^ day^−1^	[31]
*λ*_*TαM*1_	Production rate of TNF-*α* by M1 macrophages	13.91	IU cell^−1^ day^−1^	[31]
*λ*_*I*1*βM*1_	Production rate of IL-1*β* by M1 macrophages	0.1022	IU cell^−1^ day^−1^	Est. from [31]
*λ*_*I*10*M* 2_	Production rate of IL-10 by M2 macrophages	0.02	IU cell^−1^ day^−1^	[31]
*λ*_*I*4*T* 2_	Production rate of IL-4 by Th2 cells	0.0775	IU cell^−1^ day^−1^	[31]
*λ*_*I*4*M* 2_	Production rate of IL-4 by M2 macrophages	0.3094	IU cell^−1^ day^−1^	[31]
*λ*_*IαM* 2_	Production rate of IFN-alpha by M2 macrophages	1 × 10^−5^	IU cell^−1^ day^−1^	[31]
*λ*_*Treg*_	Production rate of Tregs from Th0 cells due to TGF-*β*	0.001	day^−1^	Est.
*λ*_*MI*10_	Production rate of M2 macrophages due to IL-10	1 × 10^−3^	day^−1^	Est. from [31]
*λ*_*I*1*βT*1_	Production rate of IL-1beta by Th1 cells	0.1022	IU cell^−1^ day^−1^	Est. from [31]
*λ*_*TαT*1_	Production rate of TNF-alpha by Th1 cells	13.91	IU cell^−1^ day^−1^	Est. from [31]
*λ*_*I*10*T*2_	Production rate of IL-10 by Th2 cells	0.02	IU cell^−1^ day^−1^	Est. from [31]
*λ*_*I*12*T*1_	Production rate of IL-12 by Th1 cells	0.13	IU cell^−1^ day^−1^	Est. from [31]
*d*_*M*1_	Death rate of M1 macrophages	0.02	day^−1^	[31]
*d*_*M* 2_	Death rate of M2 macrophages	0.008	day^−1^	[31]
*d*_*T*1_	Death rate of Th1 cells	1.97 × 10^−1^	day^−1^	[31]
*d*_*T*2_	Death rate of Th2 cells	1.97 × 10^−1^	day^−1^	[31]
*d*_*Iγ*_	Degradation rate of IFN-*γ*	2.16	day^−1^	[31]
*d*_*Tα*_	Degradation rate of TNF-*α*	55.45	day^−1^	[31]
*d*_*I*1*β*_	Degradation rate of IL-1*β*	6.65	day^−1^	[31]
*d*_*I* 4_	Degradation rate of IL-4	50	day^−1^	[31]
*d*_*I*10_	Degradation rate of Il-10	8.32	day^−1^	[31]
*d*_*I*12_	Degradation rate of IL-12	1.38	day^−1^	[31]
*d*_*I*α_	Degradation rate of IFN-*α*	2.16	day^−1^	Est. from [31]
*M*_0_	Constant source of monocytes	5 × 10^10^	cells L^−1^	[31]
*T*_0_	Constant source of naïve T cells	2 × 10^10^	cells L^−1^	[31]
*K*_*T*1_	Th1 cell saturation	1 × 10^10^	cells L^−1^	[31]
*K*_*Iγ*_	IFN-*γ* saturation	2.6 × 10^6^	IU L^−1^	[31]
*K*_*I* 2_	IL-2 saturation	8 × 10^6^	IU L^−1^	[31]
*K*_*I* 4_	IL-4 saturation	2.6 × 10^6^	IU L^−1^	[31]
*K*_*I*10_	IL-10 saturation	3 × 10^4^	IU L^−1^	Est. from [31]
*K*_*I*12_	IL-12 saturation	1.95 × 10^8^	IU L^−1^	[31]
*K*_*M*1_	M1 saturation	5 × 10^10^	cells L^−1^	[31]
*K*_*M* 2_	M2 saturation	1 × 10^11^	cells L^−1^	[31]
*K*_*I*1*β*_	IL-1*β* saturation	1.3 × 10^5^	IU L^−1^	[31]
*K*_*Iα*_	IFN-*α* saturation	2.6 × 10^6^	IU L^−1^	Est. from [31]
*K*_*Tα*_	TNF-*α* saturation	1.3 × 10^7^	IU L^−1^	[31]
*K*_*TGF β*_	TGF-beta saturation	2600	IU L^−1^	Est. from [31]
*g*_*TGF β*_	Concentration of TGF-*β* for half-maximal activation of Tregs	7 × 10^4^	IU L^−1^	Est.
*δ*_*M*1_	Death rate of cancer cells by M1 macrophages	1 × 10^−9^	L cell^−1^ day^−1^	Est. from [32]
*δ*_*T*1_	Death rate of cancer cells by Th1 cells.	1 × 10^−9^	L cell^−1^ day^−1^	Est. from [32]
*T*_*I*10_	IL-10 concentration that reduces CTL anti-tumor response by half	3 × 10^4^	IU L^−1^	Est.
*η*_*AI*10_	Degradation rate of anti-IL-10	3.3 × 10^−2^	day^−1^	Est. from [56]
*b*_4_	Rate of loss of free IL-10 due to binding with anti-IL-10	15	L mg^−1^ day^−1^	Est. from [56]
*b*_5_	Rate of loss of free anti-IL-10 due to binding with IL-10	1.665 × 10^−12^	L IU^−1^ day^−1^	Est. from [56]
